# The Assessment and Treatment of Post-traumatic Stress Disorder in Autistic People: A Systematic Review

**DOI:** 10.1007/s40489-024-00430-9

**Published:** 2024-02-21

**Authors:** Alice M. G. Quinton, Dorota Ali, Andrea Danese, Francesca Happé, Freya Rumball

**Affiliations:** Social, Genetic & Developmental Psychiatry Centre, Institute of Psychiatry, Psychology and Neuroscience, King’s College London, London, UK

**Keywords:** Autism, Autism spectrum, Trauma, Post-traumatic stress disorder, Trauma treatment

## Abstract

Adverse life events and mental health conditions are unfortunately common amongst autistic adults and children; this may present a vulnerability to developing post-traumatic stress disorder (PTSD). This systematic review provides an update of Rumball’s (*Review Journal of Autism and Developmental Disorders*, *6*, 294–324, 2019) systematic review of PTSD in autistic individuals and identifies 18 new studies published from 2017 to 2022, reflecting increased research interest in PTSD in autistic populations. Included literature suggests that autistic adults and children experience more severe PTSD symptoms compared to their non-autistic peers, with at least comparable rates of occurrence. We provide a comprehensive overview of this emerging field and identify the need for future research to validate PTSD symptom assessment tools and treatment strategies and investigate unique manifestations of trauma-related symptoms in autistic individuals.

Autism spectrum disorder (ASD) is defined by difficulties with social communication, repetitive behaviours, and restricted interests (American Psychiatric Association, 2013). Autistic children and adults have higher prevalence of mental health conditions when compared to non-autistic peers (Kerns et al., 2020a, b), with particularly high rates of anxiety disorders. Formerly classified as an anxiety disorder, post-traumatic stress disorder (PTSD) is a psychiatric disorder that can develop after experiencing a traumatic event. PTSD is characterised by persistent re-experiencing of the traumatic event, such as flashbacks and intrusive memories, as well as avoidance, negative appraisal, and hypervigilance to threat (Smith et al., 2019). If untreated, PTSD can have a detrimental impact on a person’s life, work, and relationships. It is therefore essential that clinicians, researchers, and society have clarity on the conceptualisation, prevalence, assessment, and support for PTSD in neurodiverse populations.

Autistic people are at heightened risk of experiencing traumatic events, adverse childhood experiences (ACES), bullying (Hoover & Kaufman, 2018; Kerns et al., 2015), stigma, and discrimination (Han et al., 2022; Turnock et al., 2022). Because of the greater exposure to triggering events, autistic people could also have a greater likelihood of developing trauma-related symptoms compared to non-autistic people (Kerns et al., 2015). Until recently, PTSD has arguably been neglected in studies of mental health in autistic populations, despite multiple factors that may contribute to autistic people’s likelihood of developing the disorder. Alongside considering societal factors and trauma exposure risk, it is possible that some cognitive and sensory characteristics associated with, but not necessarily inherent to, being autistic, such as alexithymia (difficulty identifying own emotions) and cognitive inflexibility, may make autistic people more susceptible to trauma-related symptoms or their maintenance. Despite this, research on autism-specific expressions of post-traumatic stress, and treatment adaptations for post-traumatic stress psychopathology, is lacking (Peterson et al., 2019).

Publications on PTSD within autistic groups recently increased, and several narrative reviews have been conducted (Haruvi-Lamdan et al., 2018; Hoover, 2020; Lobregt-van Buuren et al., 2021; Peterson et al., 2019). These reviews highlight the overlap of pathways that potentially might connect autism and PTSD, in particular, emotion regulation and sensory sensitivities, and propose that autism may lead to ‘diagnostic overshadowing’ of PTSD symptoms in clinical contexts—when all difficulties experienced by an autistic person are attributed to autism leading to missed PTSD diagnoses.

A previous systematic review of studies of PTSD in autism between 1980 and 2017 (Rumball, 2019) called for large-scale, well-controlled, gender-balanced studies using multi-informant and epidemiological data to assess the effects of trauma and the prevalence and risk of PTSD in autistic individuals compared to the general population. The review collated available data to produce estimates of PTSD prevalence in autistic adults and children, which were the same or higher in autistic people than in the general population. However, to date, there is no epidemiological population-based estimate for prevalence of PTSD in the autistic population. The review identified a need for investigations into different presentations of PTSD in autistic children and adults and the unique impact of trauma and PTSD on core autistic traits. Rumball (2019) concluded that for PTSD diagnosis and treatment, assessment tools are needed that can appropriately distinguish between characteristics of autism and symptoms of PTSD, and that validation of PTSD treatments in autistic people is crucial.

Here, we have sought to update the work by Rumball (2019) to provide a systematic overview of this emerging field.

## Methods

### Search

This systematic review was registered with PROSPERO (ref: CRD42021293550) and conducted in accordance with PRISMA guidelines (Page et al., 2021). The search for studies published in the English language since the termination of Rumball’s (2019) systematic review search (May 2017), up until January 2023, was conducted in PubMed, Web of Science, Embase, Scopus, Cochrane Library, PILOTS, PsychInfo, and Medline.

We followed the inclusion and exclusion criteria described in Rumball (2019). The following search terms were used: ‘autis*’, ‘asperger*’, ‘Pervasive developmental disorder’, ‘PDD-NOS’, ‘childhood disintegrative disorder’, in combination with: ‘posttraumatic stress disorder’, ‘post-traumatic stress disorder’, ‘post traumatic stress disorder’, ‘PTSD’, ‘acute stress disorder’, ‘acute stress reaction’, or ‘trauma*’. Publications were considered for inclusion in the full-text analysis if they reported primary data pertaining to PTSD assessment, prevalence, or treatment in one or more individuals of any age with a diagnosis of ASD. We included studies with cross-sectional, longitudinal, experimental, case series, and randomised controlled trial designs. We excluded reviews, meta-analyses, theoretical articles, and grey literature (e.g., conference abstracts, theses, and book chapters). Studies that did not use a formal diagnosis of PTSD or a clinically significant score on a standardised diagnostic scale for PTSD were excluded. We excluded any study that did not include individuals with a confirmed diagnosis of an ASD, which included Asperger’s disorder/syndrome, autistic disorder, childhood autism, atypical autism, pervasive developmental disorder not otherwise specified (PDD-NOS), and childhood disintegrative disorder.

All titles and abstracts were first imported into Endnote, duplicates were then removed, and an initial screening of titles and abstracts was conducted. The entire search and screening process was conducted independently by AQ and DA. Any disagreements over eligibility were discussed and resolved between the reviewers and FR.

### Data Extraction

Data on sample characteristics, assessment, prevalence, presentation of PTSD (method of PTSD diagnosis, proportion of PTSD cases in the autistic sample, outcome of the assessment, symptom presentation, and/or comparisons across groups), and PTSD treatment (treatment method, treatment outcome measure, time points for outcome measurement, and outcome of treatment) were extracted for group studies (e.g., cohort or case–control studies) and case studies. Extraction was conducted by both AQ and DA, and narrative synthesis was conducted by AQ.

To collate data on PTSD prevalence, studies were classified as reporting current or lifetime PTSD in children and adolescents (under 18 years of age) or in adults (18 years and older). If the study included a range of ages, the mean age was used to classify it. Within these age groups, studies were collated separately according to their method of PTSD assessment, collating together those that reported rates of diagnosis given by a clinician or diagnostic interview and those that used questionnaire cut-off thresholds (e.g., PCL-5).

### Quality Assessment

Observational cohort and case–control studies were assessed using a modified version of the Newcastle–Ottawa Scale (Wells et al., 2012), and cross-sectional studies were assessed using Joanna Briggs Institute (JBI) checklists (https://jbi.global/critical-appraisal-tools). Where available, we assessed and reported the method of autism and/or PTSD diagnosis in all studies. Any disagreement in quality assessment was discussed by AQ and DA. If no resolution was reached, FR and FH were consulted. Studies were not excluded from synthesis on the basis of the quality assessment.

## Results

### Study Selection

**Fig. 1:**
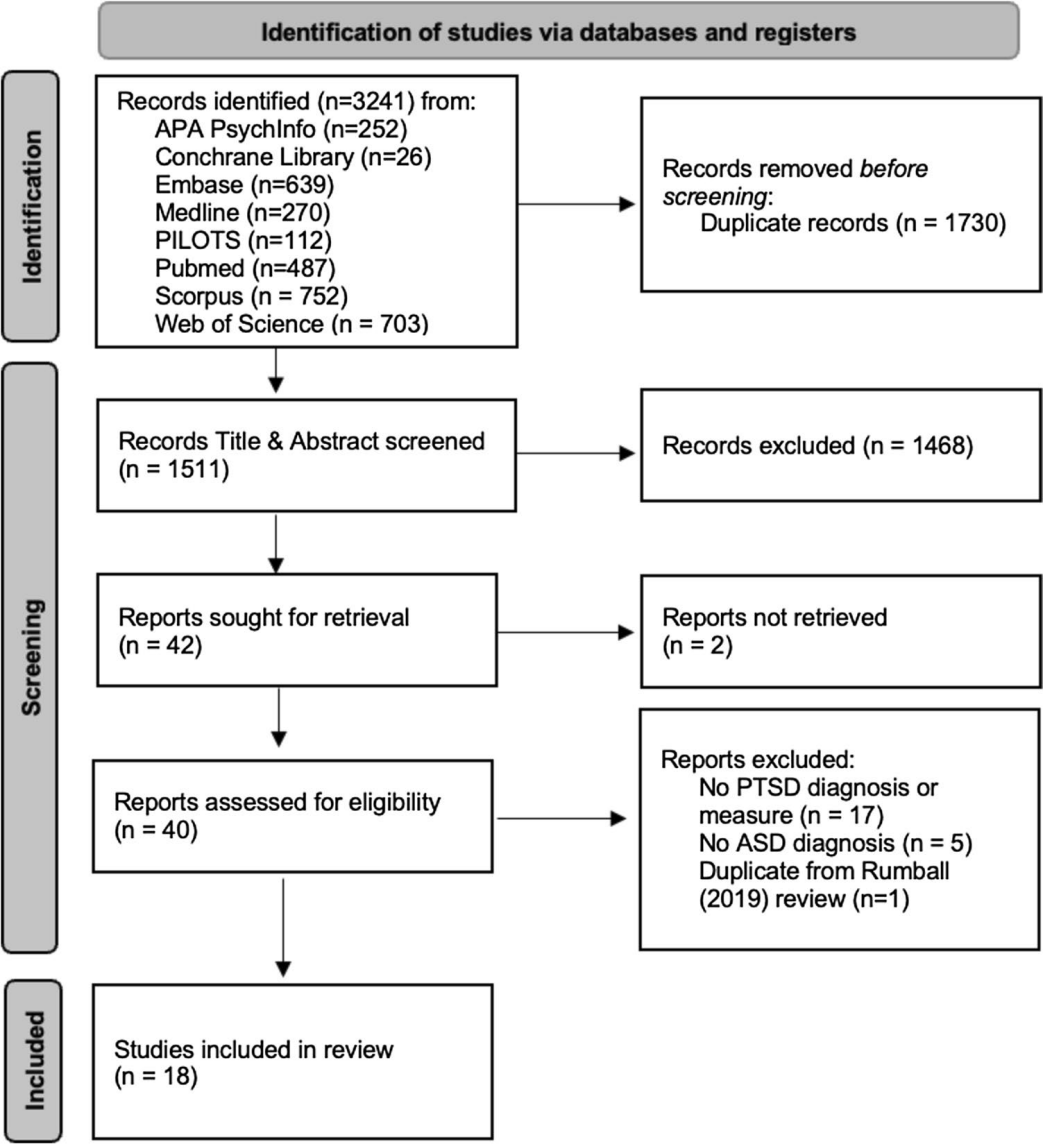
PRISMA flow diagram for systematic reviews

The search returned 3241 publications, 1730 of which were duplicates, leaving 1511 papers for title and abstract screening. At the stage of title and abstract screening, 1668 papers were excluded (here, the inter-rater reliability was 72.5%), leaving 40 papers for full-text screening. The excluded papers at the title and abstract screening stage were either not the desired publication type (reviews, grey literature, letters to the editor, and conference posters), not in populations with professionally diagnosed autism (for example, participants were parents or professionals working with autistic people), did not concern the assessment of PTSD in autistic individuals, or were not in English. From the remaining 40 papers that were retrieved for full-text screening, 18 studies met the inclusion criteria (inter-rater reliability was 86.5%). Full details of study selection can be seen in the PRISMA flow diagram (Fig. 1), and study characteristics for the final 18 papers are detailed in Table 1. Results are described below, split into assessment, rates, and treatment of PTSD in autistic people.

**Table 1:** Study Characteristics

Author (year)	Title	Location	Study type	Reason for referral (case reports) or sampling technique (group studies)	*N* ASD participants	*N* comparison group	Age group	Mean age in years (range)	Female % or sex or gender	ASD method of diagnosis	IQ level	Trauma types
Bitsika and Sharpley (2021)	Direct and inverse correlates of post-traumatic stress disorder among school-age autistic boys	Australia	Survey	Recruited via calls for participation made to parent organisations. Parents were not restricted to mothers	71	0	C&A	11.63 (7–18)	Male	Formal diagnosis from psychiatrist confirmed by psychologist	IQ at least 70 (assessed by Wechsler individualised test)	Bullying
Brenner et al. (2018)	Behavioural Symptoms of Reported Abuse in Children and Adolescents with Autism Spectrum Disorder in Inpatient Settings	USA	Clinical sample with and without abuse histories	Recruited via six psychiatric hospitals as part of the autism inpatient collection study	350	None; within ASD group compared those with abuse histories (*N* = 99) to those with no reported abuse (*N* = 251)	C&A	All: 12.9 (4–21) Reported abuse (*N* = 99): 12.89 No reported abuse (*N* = 251): 12:88	All: 21% Reported abuse: 26.3% No reported abuse: 19.5%	SCQ (score > 12) or were referred by clinical team. All participants did ADOS-2	42% of sample fell below IQ cut-off of 70 for ID, and 32% had very low verbal ability as measured by ADOS-2 (Module 2)	Physical, sexual, or emotional abuse *Of those (N* = *99) with reported abuse histories: 13% physical abuse, 12% emotional abuse, 8% sexual abuse, 1% sexual & physical, 1% sexual & emotional, 16% physical & emotional*
Carmassi et al. (2019)	Is There a Major Role for Undetected Autism Spectrum Disorder with Childhood Trauma in a Patient with a Diagnosis of Bipolar Disorder, Self-Injuring, and Multiple Comorbidities?	Italy	Case report	Admitted as inpatient to psychiatric unit with depressive symptomology, rumination, anxiety, alteration in neurovegetative pattern and suicidal ideation. Diagnosis of ASD and childhood trauma emerged during this hospitalisation. Comorbidities included Type II bipolar, binge eating, and panic disorder	1	0	Adult	35	Female	AQ (38/50), RAADS-R (146/240), AdAS Spectrum (99/160)	Average/above average IQ	Sexual abuse (Repeated childhood); loss of work, family and pet deaths, victim of a crime
Fazel et al. (2020)	Five Applications of Narrative Exposure Therapy for Children and Adolescents Presenting With Post-Traumatic Stress Disorders	UK	Case report	In-patient at adolescent psychiatric unit presenting with psychosis. PTSD was determined to be the main driver behind her psychotic symptoms	1	0	C&A	15	Female	Not reported	Borderline ID with difficulties with expressive and receptive language	Physical and sexual abuse
Golan et al. (2022)	The comorbidity between autism spectrum disorder and post-traumatic stress disorder is mediated by brooding rumination	Israel	Case control study	Recruited via internet forums, social networks, and community programmes for autistic adults	34	66	Adult	Autistic group = 23.29; non-autistic group = 23.02	Autistic group = 41.2%; non-autistic group = 43.9%	DSM diagnosis assessed by psychiatrist or clinical psychologist, confirmed by research team using DSM checklist. AQ used to screen comparison group	Average intellectual ability	In autistic group: 45.6% social, 14.7% physical assault, 10.3% sexual assault, 5.9 serious medical condition, 3.1% each for unexpected death, domestic violence, and other stressful life events
Haruvi-Lamdan et al. (2020)	Autism spectrum disorder and post-traumatic stress disorder: an unexplored co-occurrence of conditions	Israel	Case control study	Recruited via internet forums and social networks, and NGOs operating community programmes for autistic adults. In control group, 12% had ADHD and 4% dep/anx	25	25	Adult	Autistic group = 22.88; non-autistic group = 22.76. Whole sample = 22.82 (18–35)	40% females in each group	Formal diagnosis from psychiatrist or clinical psychologist	Excluded ID	Range of traumas assessed using the LEC-5; sexual assault, exposure to war, serious accident, life-threatening illness, bullying
Hoch and Youssef (2020)	Predictors of Trauma Exposure and Trauma Diagnoses for Children with Autism and Developmental Disorders Served in a Community Mental Health Clinic	USA	Community sample	Recruited from provider of community services for autism, developmental disorders, and mental illness (*N* = 7695)	3744	Trauma exposed children with developmental disorders (*N* = 95) and children with mental health issues (*N* = 1261)	C&A	7.46 (0.6–17.83)	22.94%	Diagnostic assessments conducted by psychologist or mental health professional using observations and ADOS2	Not reported for autistic group	Criteria A type traumas
Hoover and Romero (2019)	The Interactive Trauma Scale: A Web-Based Measure for Children with Autism	USA	Diagnosis	Recruited from outpatient behavioural treatment centres	20	0	C&A	11 (8–14)	43%	Documented medical diagnosis of ASD	PVT—mean score: 97.3. No specific verbal level was used for inclusion	≥ 1 potentially traumatic event reported in clinic. Child maltreatment or other violence, peer victimisation, witnessing interpersonal violence, or traumatic loss
Kildahl and Jørstad (2022)	Post-traumatic stress disorder symptom manifestations in an autistic man with severe intellectual disability following coercion and scalding	Norway	Case report	Referred for assessment of problematic avoidance and ‘challenging’ behaviour	1	0	Adult	50 s	Male	Diagnosed as a toddler, previously thoroughly assessed	Severe ID (Vineland Adaptive Behaviour Scale results were in line with this diagnosis)	Physical coercion into undergoing medical examination, caregiver negligence—second degree burns from hot shower
Kupferstein (2018)	Evidence of increased PTSD symptoms in autistics exposed to applied behaviour analysis	USA	Survey	Half of participants Interactive Autism Network (IAN) Research database, the rest recruited through social media, gatherings, social skills groups, and support groups	460 (243 autistic adults; 217 children reported by caregivers)	Within-group comparison—between applied behaviour analysis and other interventions	All ages	1–73	55% female adults; 21% female children	Self or parent report of formal diagnosis or self-diagnosis	Not reported	Therapeutic intervention; applied behavioural analysis
Kupferstein (2020)	Why caregivers discontinue applied behavior analysis (ABA) and choose communication-based autism interventions	USA	Online Survey	Recruited from the Interactive Autism Network (IAN) Research database, social media, gatherings, social skills groups, and support groups	460 (218 caregivers of children and 242 autistic adults)	0	All ages	Not reported	Not reported	Self or parent report of formal diagnosis or self-diagnosis	Not reported	Therapeutic intervention; applied behavioural analysis
Lobregt-van Buuren et al. (2019)	Eye Movement Desensitization and Reprocessing (EMDR) Therapy as a Feasible and Potential Effective Treatment for Adults with Autism Spectrum Disorder (ASD) and a History of Adverse Events	Netherlands	Non-randomised add-on study	Recruited from outpatient service of mental health institutes sand ASD specialist clinics	21	N/A	Adult	34.48	38.10%	DSM-IV diagnosis by experienced clinician, using interviews, ADI-R, and information from schools and other psychiatric services	Estimated IQ > 80 (based on education)	Physical or sexual abuse from a family member, witnessing violence between parents, death of a relative, suicide attempt of a parent, suicide attempt as a child, assault/rape, bullying, divorce (parents or partner), adultery of mother, adverse treatment at a hospital as a child, experiencing a crime, and ‘emotional mismatch of parents and child’
Paul et al. (2018)	Victimisation in a French population of children and youths with autism spectrum disorder: A case control study	France	Case control study	Recruited from a specialised diagnostic centre	39	53	C&A	Autistic group = 13.23 (8–18); non-autistic group = 12.82 (7.6–18)	Autistic group = 15.4%; non-autistic = 15.1%	Does not report. Recruited from a specialist diagnostic centre, so assumed professional	Average/above average IQ	Maltreatment, ‘conventional crime’, sexual, relational, witnessed as measured by JVQ
Reuben et al. (2021)	Interpersonal Trauma and Posttraumatic Stress in Autistic Adults	USA	Online survey	Autistic adults—self reported medical or suspected diagnosis and > 65 score on RAADS-R—recruited from online communities frequented by autistic individuals	687		Adult	Ages: < 18 years old 18 to 21–28% 22 to 30–36% 31 to 40–22% 41 + –12%	37% cis women 2% trans women 35% cis men 6% trans men19% non-binary or other	Self-identified formal or suspected diagnosis. Scored above ≥ 65 on RAADS-R screener. 66% self-reported professional diagnosis	Not reported	Range of traumas assessed using the LEC-5. 72% has experienced physical, sexual, or other unwanted sexual experience. On average, participants are exposed to 8.05 types of trauma
Reuben et al. (2022)	PTSD in autistic adults: Correlates of meeting DSM-5 criteria and predictors of professional diagnosis	USA	Online survey		677	0	Adult	39 (19–67) < 18 years old	37% cis women 2% trans women 35% cis men 6% trans men 19% non-binary or other	Autistic adults self-reported medical or suspected diagnosis and scored > 65 score on RAADS-R Recruited from online communities frequented by autistic people	Not reported	Range of traumas assessed using the LEC-5. In those self-reporting, a formal PTSD diagnosis (*N* = 134), traumas reported included interpersonal trauma (94%), physical assault (80%), sexual assault (68%), and other unwanted sexual experience (86%)
Rumball et al. (2020)	Experience of Trauma and PTSD Symptoms in Autistic Adults: Risk of PTSD Development Following DSM-5 and Non-DSM-5 Traumatic Life Events	UK	Online survey	Recruited via researchers and clinicians in NHS adult autism and mental health services, university participant databases, and wider advertising	59	0	Adult	39 (19–67)	61.02%	Self-identified formal diagnosis (DSM or ICD criteria). This was additionally confirmed using NHS record in 48/59 cases	Average/above average; ID excluded	Range of traumas assessed using the LEC-5. DSM-5 Criterion A events (*N* = 18) and non-DSM-5 life events (*N* = 20), or both (*N* = 15)
Rumball et al., (2021a)	Co-occurring mental health symptoms and cognitive processes in trauma-exposed ASD adults	UK	Online Survey	Recruited from NHS clinic, ASD recruitment lists, online and via local charities and activity centres. Conducted online (*n* = 55), by post (*n* = 3) or in person (*n* = 1)	59	0	Adult	39 (19–67)	61.02%	Self-identified as having formal diagnosis (DSM or ICD criteria). This was additionally confirmed using NHS records in 48/59 cases	Average/above average. ID excluded; level of functioning was not formally assessed	LEC-5 and ASD-specific trauma questions. Qualitative reports showed worst trauma was a DSM-5 criteria A event (*N* = 33) or not (*N* = 35)
Rumball et al., (2021b)	Heightened risk of posttraumatic stress disorder in adults with autism spectrum disorder: The role of cumulative trauma and memory deficits	UK	Case control study	Recruited via social media, ASD charities, activity centres and research participant lists	38	44	Adult	Autistic group = 32.9 (18–68); non-autistic group = 31.4 (19–57)	Autistic group = 57.9%; non-autistic group = (autistic group); 70.5% (non-autistic group)	Self-reported formal clinical diagnosis of ASD	Not reported	Range of traumas assessed using the LEC-5, and asked about ‘other’ trauma

*AdAS Spectrum*, Adult Autism Subthreshold Spectrum; *ADHD*, attention deficit hyperactivity disorder; *ADOS-2*, Autism Diagnostic Observation Schedule-Second Edition; *AQ*, autism quotient; *ASD*, autism spectrum disorder; *C&A*, child and adolescent; *dep/anx*, depression and/or anxiety; *DSM*, Diagnostic and Statistical Manual of Mental Disorders; *ICD*, World Health Organization’s International Classification of Diseases; *ID*, intellectual disability; *JVQ*, Juvenile Victimisation Questionnaire; *LEC-5*, Life Events Checklist for DSM-5; *NGO*, nongovernmental organisation

### Assessment of PTSD in Autistic People

All 18 studies reported information on the assessment of PTSD in autistic people, 10 were cross-sectional group studies (Bitsika & Sharpley, 2021; Brenner et al., 2018; Hoch & Youssef, 2020; Hoover & Romero, 2019; Kupferstein, 2018, 2020; Reuben et al., 2021, 2022; Rumball et al., 2020, 2021a), four were case–control studies (Golan et al., 2022; Haruvi-Lamdan et al., 2020; Paul et al., 2018; Rumball et al., 2021b), three were case studies (Carmassi et al., 2019; Fazel et al., 2020; Kildahl & Jørstad, 2022), and one was a non-randomised add-on study where participants were their own controls (Lobregt-van Buuren et al., 2019). Details of each study are shown in Table 2.

**Table 2:** Outcomes for the included studies

Study		Assessment, prevalence, and presentation of PTSD				Treatment of PTSD			
Author	Title	PTSD method of diagnosis (interview or questionnaire)	No. autistic participants (*n*)	Proportion of PTSD cases in autistic sample (%)	Outcome of assessment: symptom presentation/comparisons across groups	PTSD treatment method (*n* sessions)	Treatment outcome measure	Treatment outcome assessed at	Outcome of treatment
Bitsika and Sharpley (2021)	Direct and inverse correlates of post-traumatic stress disorder amongst school-age autistic boys	CASI-4R PTSD Subscale—an indication of PTSD rather than a formal diagnosis of PTSD	71	N/A	Parents rating of son’s difficulty socialising correlated with CASI-4R PTSD Scores Significant correlation between PTSD symptoms and experience of bullying (80% of sample have been bullied); bullied boys had significantly higher CASI-4R PTSD scores than those who were not	N/A	N/A	N/A	N/A
Brenner et al. (2018)	Behavioural Symptoms of Reported Abuse in Children and Adolescents with Autism Spectrum Disorder in Inpatient Settings	PTSD diagnosis from inpatient treatment team. PTSD-specific items from CASI-5, with additional items selected by lead authors based on the DSM-5 criterion. If > 1 item represented a DSM-5 criterion, the items were averaged. Caregivers were also asked if child had history of abuse	350	7 (2%)	Comparing participants with clinical PTSD diagnosis to those with caregiver-reported abuse histories without a clinical PTSD diagnosis; participants with PTSD had more intrusive thoughts, distressing memories, persistent fear, and temper tantrums	N/A	N/A	N/A	N/A
Carmassi et al. (2019)	Is There a Major Role for Undetected Autism Spectrum Disorder with Childhood Trauma in a Patient with a Diagnosis of Bipolar Disorder, Self-Injuring, and Multiple Comorbidities?	TALS-SR and RRS	1	1 (100%)	Lifetime post-traumatic stress symptoms; particularly re-experiencing and maladaptive coping. They suggest ruminations link ASD and PTSD; as prevents processing of traumatic experience	Tailored CBT psychotherapy focused on trauma elaboration *Medications:* Lithium mood stabiliser, sertraline 50 mg antidepressant, aripiprazole 5 mg for ‘behavioural disturbances’	N/A	N/A	Showed significant clinical global improvement after sertraline and aripiprazole in hospital. Does not report outcome of trauma-focused therapy
Fazel et al. (2020)	Five Applications of Narrative Exposure Therapy for Children and Adolescents Presenting With Post-Traumatic Stress Disorders	Not reported, diagnosis made in psychiatric hospital	1	1 (100%)	Difficulty with social interactions had contributed to multiple traumas (majority at school). She struggled to see different perspectives about events and had poor recall and recognition difficulties. Low ability for new learning was believed to contribute to poor encoding of event	Narrative exposure therapy (NET) (9–10 sessions)	Observation/reports by staff on the ward		Reduction in self-harm, aggressive incidents, and relieving symptoms
Golan et al. (2022)	The comorbidity between autism spectrum disorder and post-traumatic stress disorder is mediated by brooding rumination	PCL-5 (cut off > 38); RRS	34	11 (32.40%)	Compared to non-autistic group, the autistic group had significantly higher proportion of participants with above threshold scores (> 38) on the PCL-5 and significantly higher PTSD and brooding rumination scores	N/A	N/A	N/A	N/A
Haruvi-Lamdan et al. (2020)	Autism spectrum disorder and post-traumatic stress disorder: an unexplored co-occurrence of conditions	LEC-5, PCL-5 cut off score ≥ 33; RRS	25	8 (32%)	Compared to non-autistic group (*n* = 1 met cut-off score), autistic individuals had significantly higher post-traumatic stress symptoms, re-experiencing and hyperarousal, and negative mood alterations	N/A	N/A	N/A	N/A
Hoch and Youssef (2020)	Predictors of Trauma Exposure and Trauma Diagnoses for Children with Autism and Developmental Disorders Served in a Community Mental Health Clinic	Diagnosis of PTSD (ICD or DSM) extracted from electronic medical record system	3744	159 (4.25%)	Diagnostic regression showed autistic children least likely to have a trauma diagnosis when compared to children with developmental disorder (14.83%) and mental health problems (29.81%)	N/A	N/A	N/A	N/A
Hoover and Romero (2019)	The Interactive Trauma Scale: A Web-Based Measure for Children with Autism	ITS prototype development; UCLA post-traumatic stress disorder reaction index for DSM-5. ITS was written to assess five DSM-5 PTSD domains and showed convergent validity with child self-report (SR) and parent report (PR) versions of the UCLA. Post-traumatic stress disorder reaction index for DSM-5	20	5 (25%)	Participants who screened positive for PTSD on ITS had significantly more trauma exposures than those who did not. 3/5 also screen positive for PTSD on UCLA-SR and 4/5 for UCLA-PR	N/A	N/A	N/A	N/A
Kildahl and Jørstad (2022)	Post-traumatic stress disorder symptom manifestations in an autistic man with severe intellectual disability following coercion and scalding	Outpatient assessment by three experienced mental health professionals (clinical psychologist, psychiatrist, and ID nurse)	1	1 (100%)	Continuously anxious, hyperarousal, hypervigilance, panic attacks, avoidance of cars or buses or bathrooms, negative cognitions/mood, triggered by hospital wear and running water, sleep difficulties, and suspected nightmares. Hitting and kicking when made to do activities outside his apartment. Difficult to ascertain re-experiencing due to limited verbal ability	Exposure-based intervention (placing desirable objects in place associated with trauma). Trauma-informed care (sensitivity to man’s communication style and focus on autonomy, safety, empowerment, and validation)	Observations		Discontinued due to it exacerbating symptoms. Reduction of distress, problematic avoidance, panic attacks
Kupferstein (2018)	Evidence of increased PTSD symptoms in autistics exposed to applied behaviour analysis	26-question survey modelled on PCL-5 with intervention-related questions. CAPS-5 severity conventions were used to score survey responses	460	212 (46% of the ABA-exposed respondents met the diagnostic threshold for PTSD)	Extreme levels of severity of PTSS were recorded in 47% of the ABA-affected subgroup	N/A	N/A	N/A	N/A
Kupferstein (2020)	Why caregivers discontinue applied behaviour analysis (ABA) and choose communication-based autism interventions	Online questionnaire (26-questions) modelled on PCL-5. Severity of symptoms was scored by individual symptom clusters, and classified by thresholds of moderate, severe, or extreme	460	193 (42% of those exposed to ABA had PTSS)	PTSS was higher in ABA that ACC interventions or no intervention at all	N/A	N/A	N/A	N/A
Lobregt-van Buuren et al., 2019	Eye Movement Desensitization and Reprocessing (EMDR) Therapy as a Feasible and Potential Effective Treatment for Adults with Autism Spectrum Disorder (ASD) and a History of Adverse Events	IES-R	21	14 (66.6%)		Eye Movement Desensitisation and Reprocessing (EMDR) (*n* = 8). Added to treatment as usual which included psychoeducation, counselling, pharmacotherapy, job coaching, housekeeping help, or case management	Adapted Anxiety Disorders Interview Schedule-Children (ADIS-C) section PTSD, the Impact of Events Scale-Revised (IES-R), BSI, and SRS-A	T1: Baseline T2: After 6–8 weeks of TAU T3: After 8 sessions of EMDR T4: Follow up at 6–8 weeks	ADIS-C and IES-R score decreased significantly after EMDR sessions and remained stable at 6–8 weeks follow up
Paul et al. (2018)	Victimisation in a French population of children and youths with autism spectrum disorder: A case control study	Interview; questionnaire—JVQ; PCL-S cut off score ≥ 33	39	3 (7.7%)	For subjects victimised at least once in their lifetime, PCL-S scores were significantly higher in autistic young people	N/A	N/A	N/A	N/A
Reuben et al. (2021)	Interpersonal Trauma and Posttraumatic Stress in Autistic Adults	PCL-5 with cut-off > 33	687	297 (43.23%)	Odds of meeting PTSD criteria and levels of dissociation were higher for those who experienced interpersonal trauma. Cisgender women and gender minorities more likely to experience trauma and meet criteria for PTSD. There was no significant difference between self-diagnosis and professional diagnosis for interpersonal trauma or PTSD	N/A	N/A	N/A	N/A
Reuben et al. (2022)	PTSD in autistic adults: Correlates of meeting DSM-5 criteria and predictors of professional diagnosis	Self-reported professional diagnosis of PTSD and the PCL-5 cut-off > 33	677	134 (20%) self report 297 (44%) PCL-5 > 33	Participants who met PCL-% cut off; worse mental health, increased impairment and lower employment. Those with PTSD diagnosis scored higher on autistic traits, mental health symptoms, and functional impairment compared to those with no diagnosis. In those who scored > 35 on PCL-5, increased post-traumatic stress and higher number of co-occurring disorders predicted professional diagnosis, whereas functional impairment negatively predicted	N/A	N/A	N/A	N/A
Rumball et al. (2020)	Experience of Trauma and PTSD Symptoms in Autistic Adults: Risk of PTSD Development Following DSM-5 and Non-DSM-5 Traumatic Life Events	PCL-5 (cut-off > 33) completed in relation to event that participant considered their worse trauma	59	28 (47%) (any trauma)	Sexual and physical abuse most common typical traumas and breakdowns were most common non-DSM5. 45% with DSM-5 trauma and 43% with non-DSM trauma met probable PTSD cut-off	N/A	N/A	N/A	N/A
Rumball et al., (2021a)	Co-occurring mental health symptoms and cognitive processes in trauma-exposed ASD adults	PCL-5 cut-off > 33. Rates of reported lifetime mental health diagnoses made in health services by a clinician, self-reported by participant	59	27 (45%) met PCL-5 cut-off and 6 (10.17%) had lifetime diagnosis of PTSD	Probable PTSD had high co-occurring mental illness and was not isolated to DSM-5 Criterion A events. Perseverative thinking and thought suppression positively correlated with PTSD anxiety and depression	N/A	N/A	N/A	N/A
Rumball et al., (2021b)	Heightened risk of posttraumatic stress disorder in adults with autism spectrum disorder: The role of cumulative trauma and memory deficits	PCL-5 (cut off > 50). Completed a PCL-5 for each LEC	38	17 (45%)	PTSD symptoms were significantly higher in autistic group; this group was also (9 times) more likely to have probable PTSD for a non-DSM trauma. PTSD at 45% in autistic group vs. 4.5% in non-autistic group	N/A	N/A	N/A	N/A

*ABA*, applied behaviour analysis; *ACC*, augmentative and alternative communication; *ASD*, autism spectrum disorder; *BSI*, Brief Symptom Inventory; *CAPS-5*, Clinician-Administered PTSD Scale for DSM-5; *CASI*, Child and Adolescent Symptom Inventory; *DSM*, Diagnostic and Statistical Manual of Mental Disorders; *ICD*, World Health Organization’s International Classification of Diseases; *ID*, intellectual disability; *JVQ*, Juvenile Victimisation Questionnaire; *LEC-5*, Life Events Checklist for DSM-5; *PCL-5*, PTSD Checklist for DSM-5; *PTSS*, post-traumatic stress symptoms; *RRS*, rumination response scale; *SRS-A*, Social Responsiveness Scale for adults; *TALS-SR*, Trauma and Loss Spectrum – Self Reported; *UCLA-SR/PR*, UCLA post-traumatic stress disorder reaction index for DSM-5 – self-report/parent report

#### Group Studies

The 15 group studies reported assessment of PTSD in a total of 5607 autistic people. Of these studies, eight reported on a total of 923 adults (Golan et al., 2022; Haruvi-Lamdan et al., 2020; Lobregt-van Buuren et al., 2019; Reuben et al., 2021, 2022; Rumball et al., 2020, 2021a, b), five reported on 4224 children (Bitsika & Sharpley, 2021; Brenner et al., 2018; Hoch & Youssef, 2020; Hoover & Romero, 2019; Paul et al., 2018), and two studies reported on the same population of 460 respondents of mixed ages (Kupferstein, 2018, 2020). All but one study (Bitsika & Sharpley, 2021), which recruited only boys, included mixed genders. Of the mixed studies, the proportion of women/girls ranged from 15.4% (Paul et al., 2018) to 61.02% (Rumball et al., 2020, 2021a).

#### Group Studies: Assessing Trauma Exposure in Autistic People

Ten of the group studies assessed participants for trauma exposure using questionnaires that were comparable with DSM-5 criteria A type traumas; six used the Life Events Checklist for DSM-5 (LEC-5) (Haruvi-Lamdan et al., 2020; Reuben et al., 2021, 2022; Rumball et al., 2020, 2021a, b), one used the Juvenile Victimisation Questionnaire (JVQ) (Paul et al., 2018), and two used similarly comprehensive lists of clinically significant traumatic life events (Golan et al., 2022; Hoover & Romero, 2019). In one study, doctors used an unstructured interview that reported negative life events as ‘stressors’ in the EMR (Hoch & Youssef, 2020); here, the trauma types listed are compatible with DSM-5 criterion A type traumas. Of these ten studies, three also assessed non-DSM-5 traumas, using the qualitative response in the LEC-5 to record ‘other’ traumatic events (Rumball et al., 2021b) or enquiring if any other event felt traumatic, caused avoidance or intrusions (Rumball et al., 2020, 2021a). One of these studies gives full details of non-DSM-5 events reported (Rumball et al., 2020), the most common of which were bullying, bereavement, and events linked to other mental health problems (e.g. ‘breakdowns’). Stress related to difficulties socialising, as well as abonnement by significant others, were also reported by multiple people. Three group studies focused on assessing post-traumatic stress as a result of a specific traumatic experience; bullying (Bitsika & Sharpley, 2021), physical, sexual, or emotional abuse (Brenner et al., 2018), and an autism intervention (Kupferstein, 2018, 2020).

In the case–control studies, autistic groups had significantly higher trauma exposure than the non-autistic groups (Haruvi-Lamdan et al., 2020; Rumball et al., 2021b), had significantly higher lifetime victimisation JVQ scores and victimisation by peers and siblings (Paul et al., 2018), and reported higher numbers of negative social events (Golan et al., 2022; Haruvi-Lamdan et al., 2020).

#### Group Studies: Assessing PTSD Symptoms in Autistic People

The studies assessed PTSD using different measurement tools. The most commonly used in studies with autistic adults was the PCL-5, which was used by eight studies to assess PTSD symptoms to indicate likely PTSD (Golan et al., 2022; Haruvi-Lamdan et al., 2020; Paul et al., 2018; Reuben et al., 2021, 2022; Rumball et al., 2020, 2021a, b). One study used the Impact of Event Scale-Revised (IES-R) and the Adapted Anxiety Disorders Interview Schedule (ADIS-C) PTSD section version for adults with mild to borderline ID (Lobregt-van Buuren et al., 2019). Two studies also asked for participants to self-report professional psychiatric diagnoses, including PTSD (Reuben et al., 2022; Rumball et al., 2021a). The five studies with autistic children used versions of the PTSD-specific items from Child and Adolescent Symptom Inventory (CASI) (Bitsika & Sharpley, 2021; Brenner et al., 2018), the UCLA post-traumatic stress disorder reaction index for DSM-5 (Hoover & Romero, 2019), and diagnostic information from clinicians either from inpatient treatment team (Brenner et al., 2018) or electronic medical records (Hoch & Youssef, 2020).

Four case–control studies assessed PTSD in groups of both autistic and non-autistic children and adults (Golan et al., 2022; Haruvi-Lamdan et al., 2020; Paul et al., 2018; Rumball et al., 2021b). These case-controlled studies found that the PTSD Checklist for DSM-5 (PCL-5) scores were significantly higher in autistic children (Paul et al., 2018) and adults (Golan et al., 2022; Haruvi-Lamdan et al., 2019; Rumball et al., 2021b) when compared to non-autistic control groups. These group differences could be biased by confounding. However, of these case–control studies, one matched the groups for sex and age (Paul et al., 2018) and three stated the control groups did not significantly differ for age, gender (Golan et al., 2022; Haruvi-Lamdan et al., 2020; Rumball et al., 2021b) or socioeconomic status (Haruvi-Lamdan et al., 2020). Two studies controlled in their analysis for significantly higher education in the non-autistic group (Golan et al., 2022; Haruvi-Lamdan et al., 2020), one of which also controlled for existing psychiatric diagnosis as this was higher in their autistic group (Golan et al., 2022). These case–control studies did not recruit on the bases of trauma exposure or match groups for level of trauma exposure (which was higher in the autistic groups).

Three studies directly compared specific PTSD symptom domains between autistic and non-autistic groups (Golan et al., 2022; Haruvi-Lamdan et al., 2020; Paul et al., 2018). None of these studies actively recruited for trauma exposure. Table 3 details which core PTSD symptoms significantly differed and which did not across these studies.

**Table 3:** Core PTSD symptoms that were compared in autistic and non-autistic participants in case–control studies

Core PTSD Symptom from DSM-5 (*Symptom name as described in paper*)	Study comparing autistic vs. non-autistic groups		
	Golan et al. (2022)	Haruvi-Lamdan et al. (2020)	Paul et al. (2018)*
Criterion B: Presence of intrusion symptoms associated with the traumatic event			
Recurrent, involuntary, and intrusive distressing memories of the traumatic event (‘*Intrusions*’, ‘*Putting back into action in games or activities*’)	A = N		A = N
Recurrent distressing dreams (‘*Nightmares*’)			A = N
Dissociative reactions where individual feels the trauma is reoccurring. (‘*Re-experiencing*’, ‘*Flashbacks*’)		A > N	A > N
Marked physiological reactions to internal or external cues that symbolise or resemble an aspect of the traumatic event (‘*Hyper-arousal*’)	A > N	A > N	
Criterion C: Persistent avoidance of stimuli associated with the traumatic event			
Avoidance of or efforts to avoid distressing memories, thoughts, or feelings, and external reminders	A = N	A = N	A > N
Criterion D: Negative alterations in cognitions and mood associated with the traumatic event			
Inability to remember an important aspect of the traumatic event (‘*Selective amnesia of facts*’)			A = N
Persistent negative emotional state (‘*Negative alterations in mood and cognition*’ (both Golan et al. (2022) & Haruvi-Lamdan et al., (2020), ‘*sadness*’ (Paul et al. (2018))	A > N	A > N	A = N
Persistent inability to experience positive emotions (‘*Emotional anaesthesia*’)			A = N
Persistent and exaggerated negative beliefs or expectations about oneself, others, or the world (‘*Brooding rumination*’, ‘*Pessimism*’)	A > N		A = N
Markedly diminished interest or participation in significant activities (‘*Anhedonia*’)			A = N
Feelings of detachment or estrangement from others (‘*Social isolation*’)			A > N
Criterion E: Marked alterations in arousal and reactivity associated with the traumatic event			
Irritable behaviour			A = N
Problems with concentration			A > N
Sleep disturbances (‘*Insomnia*’)			A > N
Hypervigilance			A > N

This table outlines which of the DSM-5’s diagnostic criteria for PTSD were assessed by the studies comparing autistic (A) and non-autistic (N) children (Paul et al., 2018) and adults (Golan et al., 2022; Haruvi-Lamdan et al., 2020; Paul et al., 2018). Where symptoms were described using different language, or could be used as a proxy, these are included in brackets in the order the studies appear in the table. ‘A > N’ indicates that symptoms were found at higher prevalence in the autistic group compared to non-autistic group, whereas ‘A = N’ indicates no significant difference between groups. *Children

#### Group Studies: Influence of Sex or Gender on PTSD Symptoms in Autistic People

Seven studies examined the influence of sex or gender on PTSD symptoms. Three studies showed no effect of gender on symptom severity scores (Paul et al., 2018; Rumball et al., 2020, 2021b), and one reported no significant gender difference in the small number of PTSD cases (*N* = 7 (2%)) (Brenner et al., 2018). Three papers reported significantly more PTSD symptoms in women (Haruvi-Lamdan et al., 2020; Reuben et al., 2021, 2022); one study (Haruvi-Lamdan et al., 2020) reported that overall symptom severity and hyperarousal symptoms were significantly higher in autistic females than non-autistic females, a pattern not seen in the males in this study. Only two papers from Reuben and colleagues (Reuben et al. 2021***, ***2022) reported more detailed gender identity demographics and found that cisgender women and gender minorities were more likely to meet PCL-5 cut-off than cisgender men.

#### Case Studies

Three papers reported case studies of PTSD in autistic people; two case studies of autistic adults (one woman and one man) and one of an adolescent girl. The autistic man was severely intellectually disabled (ID), the adolescent girl had borderline ID, and the adult woman had average/above average IQ. Both the autistic woman and the autistic girl were inpatients at psychiatric units, presenting with other psychiatric difficulties; the autistic girl was admitted with psychosis (Fazel et al., 2020), and the autistic woman with type II bipolar, binge eating, and panic disorder (Carmassi et al., 2019). PTSD was described to be the main driver behind the young girl’s psychotic symptoms (Fazel et al., 2020). For the autistic woman, her childhood trauma and autism diagnosis were only detected during her hospitalisation for other mental health difficulties (Carmassi et al., 2019). The middle-aged man was referred to outpatient assessment for ‘problematic avoidance’ and ‘challenging behaviour’ (Kildahl & Jørstad, 2022).

Sexual abuse, amongst other traumas, had been experienced by the two autistic female participants (see Table 1) (Carmassi et al., 2019; Fazel et al., 2020). In both cases, the young girl and the adult woman were able to report their own traumas. The autistic man with ID’s traumas was reported by other informants (Kildahl & Jørstad, 2022). This was due to the man’s verbal communication skills; he did not use alternative communication strategies to communicated with caregivers using objects and some limited language (20–30 words which included names and ‘regularly used idiosyncrasies’). The man’s difficulties, such as refusing to go to the bathroom, were assessed as avoidant symptoms connected to the caregiver neglect he was reported to have experienced. This abuse included getting burnt in the shower; thus, it was presumed that his avoidance had generalised to the whole bathroom.

In terms of PTSD assessment, the case studies varied. One detailed the symptom assessment scales used to assess PTSD (Carmassi et al., 2019), one detailed that outpatient assessment was completed by three experienced mental health professionals (clinical psychologist, psychiatrist, and ID nurse) (Kildahl & Jørstad, 2022), and one did not detail how an assessment was made (Fazel et al., 2020). Given the latter participant was an inpatient at a psychiatric hospital, it may be assumed that a professional diagnosis was made. None of the studies specified if DSM or ICD diagnostic guidelines were used. PTSD symptom presentation was detailed for each patient and followed similar profiles to PTSD in the general population. Symptoms included re-experiencing and maladaptive coping (Carmassi et al., 2019), avoidance of environments linked to trauma (Fazel et al., 2020; Kildahl & Jørstad, 2022), continuous hyperarousal, negative cognitions, hypervigilance, panic attacks, sleep difficulties, and suspected nightmares (the autistic man was unable to report these himself) (Kildahl & Jørstad, 2022).

All the case studies discussed how autistic traits impacted the assessment of PTSD symptoms. For example, as the autistic man was unable to communicate verbally, the authors noted that re-experiencing symptoms and nightmares was hard to assess (Kildahl & Jørstad, 2022). Autistic difficulties that were suggested to have impacted the patient’s encoding and subsequent processing of their traumatic experience included poor memory recall, struggles seeing different perspectives, low ability for new learning (Fazel et al., 2020), and rumination (Carmassi et al., 2019).

### Rates of PTSD Diagnoses in Autistic Populations

Of the studies included, 12 studies reported incidences of PTSD; nine of these studies used symptom scales to assess if people met cut-off for probable PTSD (Golan et al., 2022; Haruvi-Lamdan et al., 2020; Hoover & Romero, 2019; Lobregt-van Buuren et al., 2019; Paul et al., 2018; Reuben et al., 2022; Rumball et al., 2020, 2021a, b), two studies had people self-report if they had a current PTSD diagnosis (Reuben et al., 2022; Rumball et al., 2021a), and two used diagnoses made by mental health professionals (Brenner et al., 2018; Hoch & Youssef, 2020). The mean sample size of the studies was 427. However, one study (Hoch & Youssef, 2020) had significantly larger numbers (*N* = 3744), as the children were recruited from a large community sample through community services for autism, developmental disorders, and mental illness. When this large sample was removed, the mean sample size was 125. Reported rates of PTSD ranged from 2 to 66.67%. The type of clinical settings the participants were seen at influenced how routinely someone was assessed for PTSD. The two studies with the lowest rates of PTSD in autistic samples were the only group studies to measure PTSD using clinician diagnosis (Brenner et al., 2018; Hoch & Youssef, 2020) and did so for children/adolescents. One of the studies used diagnostic information from an inpatient treatment team (2%) (Brenner et al., 2018) and the other used electronic medical records (4%) (Hoch & Youssef, 2020). The study with the highest proportion of PTSD diagnoses (66.67%) (Lobregt-van Buuren et al., 2019) was recruiting trauma-exposed participants to treat trauma-related symptoms with Eye Movement Desensitisation and Reprocessing (EMDR) therapy.

These 12 studies reported a total of 611 PTSD cases out of a sample of 5076 autistic individuals (41.72% female). Table 4 reports the percentage of PTSD cases when study samples are pooled and grouped by PTSD assessment method. As there were studies that selectively recruited trauma-exposed participants or those with anxiety disorders, these were subsequently removed to assess their weighting.

**Table 4:** Estimated rates of PTSD in autistic adults and children

Papers included	Review(s) scope	PTSD assessment method	Study recruitment inclusion criteria (trauma exposure, anxiety disorder, and autism only)	*N* ASD sample	*N* cases of PTSD	% rate of PTSD	Mean age	Min age	Max age
Children studies: Current PTSD									
Brenner et al. (2018); Hoch and Youssef (2020)	2017–2023	Professional/diagnostic interview	Autism only	4094	218	5.32%	10.18	0.60	21.00
Hoover and Romero (2019); Paul et al. (2018)	2017–2023	Questionnaire cut-off	All studies	59	8	13.56%	12.12	8	18
Hoover and Romero (2019)	2017–2023	Questionnaire cut-off	Trauma exposure	20	5	25.00%	11	8	14
Paul et al. (2018)	2017–2023	Questionnaire cut-off	Autism only	39	3	7.69%	13.23	8	18
Brenner et al. (2018); Hoch and Youssef (2020); Hollocks et al. (2016); McConachie et al. (2014); Reinvall et al. (2016); Storch et al. (2013); White et al. (2012, 2013); Wood et al. (2009, 2015)	1980–2023	Professional/diagnostic interview	All studies	4476	230	5.14%	11.60	0.60	21
McConachie et al. (2014); Storch et al. (2013); White et al. (2012, 2013) Wood et al. (2009, 2015)	1980–2023	Professional/diagnostic interview	Trauma exposure and anxiety disorders	267	11	4.12%	12.07	7	17
Brenner et al. (2018); Hoch and Youssef (2020); Hollocks et al. (2016); Reinvall et al. (2016)	1980–2023	Professional/diagnostic interview	Autism only	4209	219	5.20%	10.65	0.60	21
Children studies: Lifetime (including current) PTSD									
Brenner et al. (2018); de Bruin et al. (2007); Hoch and Youssef (2020); Hollocks et al. (2016); McConachie et al. (2014); Mehtar and Mukaddes (2011); Reinvall et al. (2016); Storch et al. (2013); White et al. (2012, 2013); Wood et al. (2009, 2015)	1980–2023	Professional/diagnostic interview	All studies	4639	242	5.22%	11.34	0.60	21
Brenner et al. (2018); de Bruin et al. (2007); Hoch and Youssef (2020); Hollocks et al. (2016); Mehtar and Mukaddes (2011); Reinvall et al. (2016)	1980–2023	Professional/diagnostic interview	Autism only	4372	231	5.28%	10.43	0.60	21
Adult studies: Current PTSD									
Golan et al. (2022); Haruvi-Lamdan et al. (2020); Lobregt-van Buuren et al. (2019); Reuben et al. (2022); Rumball et al., (2020, 2021a, b)	2017–2023	Questionnaire cut-off	All studies	907	399	43.99%	32.93	18.00	67.00
Golan et al. (2022); Haruvi-Lamdan et al. (2020); Reuben et al. (2022); Rumball et al., (2021b)	2017–2023	Questionnaire cut-off	Autism only	774	333	43.02%	29.50	18.00	67.00
Lobregt-van Buuren et al. (2019) Rumball et al., (2020, 2021a, b)	2017–2023	Questionnaire cut-off	Trauma exposure	133	66	49.62%	37.49	19	67
Adult Studies: Lifetime (Including current) PTSD									
Rumball et al., (2021a)	2017–2023	Professional/diagnostic interview	Trauma exposure	59	6	10.17%	39	19	67
Hofvander et al. (2009); Reuben et al. (2022); Rumball et al., (2021a); Taylor and Gotham (2016)	1980–2023	Professional/diagnostic interview	All	894	142	15.88%	31.43	16.00	67
Hofvander et al. (2009); Reuben et al. (2022); Taylor and Gotham (2016)	1980–2023	Professional/diagnostic interview	Autism only	835	136	25.00%	28.90	16.00	67

For studies reporting those who met the cut-off threshold for PTSD on symptom questionnaires, seven studies reported PTSD in 399 of 907 (43.99%, range 32–66.67%) autistic adults, and two studies reported PTSD in 8 of 59 (13.23%, range 7.69–25%) autistic children. For studies reporting professional PTSD diagnoses, two studies reported a total of 218 of 4094 children (5.32%, range 2–4.25%) had a current PTSD diagnosis, and two studies reported 140 of 736 autistic adults (19.02%, range 10.17–19.79%) had a PTSD diagnosis in their lifetime. There were 4 studies altogether that selectively recruited trauma-exposed participants (Hoover & Romero, 2019; Lobregt-van Buuren et al., 2019; Rumball et al., 2020, 2021a). This sampling strategy naturally inflated the rates of PTSD, and these studies had higher rates of people meeting PTSD cut-offs (49.62% in adults, range 45.28–66.67%; 25% in one study in children) compared to those who recruited based on autism diagnosis alone (43.02% in adults, range 32–44.74%; 7.69% in one study in children). Collating the data from these studies from 2017 onwards with those included in Rumball’s (2019) review from 1980 to 2017 (see Table 4) gives estimated rates for autistic children and adolescents reporting current (5.14%, range 0–5.88%) and lifetime (5.22%, range 0–17.39%) PTSD diagnoses and rate of autistic adults with a diagnosis of PTSD in their lifetime (15.88%, range 0–19.79%).

### Treatment of PTSD in Autistic People

Four studies investigated PTSD treatment in autistic people. Of these, three were case studies and one was a group study that utilised a non-randomised add-on design, where participants first received sessions of treatment as usual (TAU) and then received additional sessions of EMDR therapy (Lobregt-van Buuren et al., 2019).

#### Group Study

Only one study empirically tested PTSD treatment in a group of autistic people. Lobregt-van Buuren and colleagues (Lobregt-van Buuren et al., 2019) assessed the efficacy of using EMDR as an add-on therapy in a group of 21 autistic adults. The participants were recruited by clinicians if they had suspected PTSD. Inclusion criteria included a score of 4 or higher on a ‘thermometer card’ that is included in the PTSD section of the Adapted Anxiety Disorders Interview Schedule-Children (ADIS-C). This indicated participants had PTSD symptoms that were related to an adverse event. In this study, the participants were treated as their own controls: after 6–8 weeks of TAU, 8 EMDR sessions were added to TAU. PTSD symptoms were assessed at four time points. The study found that PTSD symptoms did not significantly improve after TAU but did significantly improve after 8 sessions of EMDR and remained stable at 6- to 8-week follow-up, as reflected in both the ADIS-C and IES-R scores.

#### Case Studies

Autistic people’s experience of PTSD treatment was reported in three case studies, with ages ranging from adolescence to middle age (Carmassi et al., 2019; Fazel et al., 2020; Kildahl & Jørstad, 2022). A form of psychological therapy was used in all case studies. Narrative exposure therapy (NET) (Fazel et al., 2020), adapted CBT (Carmassi et al., 2019) and trauma-informed care (Kildahl & Jørstad, 2022) were reported to be successful, while an exposure-based intervention was reported to worsen avoidance symptoms (Kildahl & Jørstad, 2022). Two studies reported the outcome of PTSD treatment on PTSD symptoms. NET resulted in reduced aggression, self-harm, and reliving symptoms after 9–10 sessions (Fazel et al., 2020). An exposure-based intervention, involving placing objects the man liked in the place he was avoiding due to the traumatic experience, was discontinued as it was reported to exacerbate his distress (Kildahl & Jørstad, 2022). Only one case study reported medication use: mood stabilisers and antidepressants were given for other psychiatric comorbidities alongside CBT for trauma elaboration and resulted in global clinical improvement (Carmassi et al., 2019). The outcome of the adapted CBT was not reported. The patient was also given aripiprazole for reported autism-related ‘behavioural disturbances’.

Two of the case studies described autism-specific adaptations made to interventions. These included taking more time with each component of the therapy process, repeated explanations of the rationale for the therapy, written prompts, conducting the sessions while walking—which provided sensory coping mechanisms and reduced dissociation (Fazel et al., 2020), and increasing staff sensitivity to specific forms of non-verbal communication (Kildahl & Jørstad, 2022﻿).

### Quality Assessment

Case–control studies were assessed using the Newcastle–Ottawa Scale (NOS) (Wells et al., 2012), and the Joanna Briggs Institute (JBI, 2016) critical appraisal tool was used for cross-sectional studies (https://jbi.global/critical-appraisal-tools). Both scales had eight items, with a maximum score of eight (stars for NOS, ‘yes’ answers for JBI). The study ratings are presented in Table 5 and 6.

**Table 5:** Quality assessment of methodology of case–control studies using Newcastle–Ottawa Scale (NOS) (Wells et al., 2012)

Study	Adequate definition of case	Representativeness of the cases	Selection of controls	Definition of controls	Comparability of cases and controls on the basis of the design or analysis	Ascertainment of exposure	Same method of ascertainment for cases and controls	Adequacy of follow-up of cohorts	Total score (max = 8)
Haruvi-Lamdan et al. (2020)	*	*	*	*	*		*		6
Rumball et al., (2021b)			*	*	*		*		4
Golan et al. (2022)	*	*	*	*	*		*		6
Paul et al. (2018)	*	*	*	*	*		*		6

^*^Indicates study meets that quality criterion

**Table 6:** Quality assessment of methodology of cross-sectional studies using Joanna Briggs Institute (JBI) critical appraisal tool Checklist for Analytical Cross-Sectional Studies (https://jbi.global/critical-appraisal-tools.html)

Study:	Were the criteria for inclusion in the sample clearly defined?	Were the study subjects and the setting described in detail?	Was the exposure measured in a valid and reliable way?	Were objective, standard criteria used for measurement of the condition?	Were confounding factors identified?	Were strategies to deal with confounding factors stated?	Were the outcomes measured in a valid and reliable way?	Was appropriate statistical analysis used?	Total Score (max = 8)
Brenner et al. (2018)	Yes	Yes	Yes	Yes	Yes	Yes	Yes	Yes	8
Hoch and Youssef (2020)	Yes	Yes	Yes	Yes	Yes	Yes	Yes	Yes	8
Hoover and Romero (2019)	Yes	Yes	Yes	No	No	No	Yes	Yes	5
Lobregt-van Buuren et al. (2019)	Yes	Yes	Yes	No	Yes	Yes	Yes	Yes	7
Rumball et al. (2020)	Yes	Yes	Yes	No	Yes	Yes	Yes	Yes	7
Rumball et al., (2021a)	Yes	Yes	Yes	No	No	No	Yes	Yes	5
Kupferstein (2018)	yes	Yes	No	No	No	No	No	Unclear	2
Kupferstein (2020)	Yes	Yes	No	No	No	No	No	No	2
Bitsika and Sharpley (2021)	*Yes*	Yes	Yes	No	Yes	Yes	Yes	Yes	7
Reuben et al. (2021)	No	Yes	Yes	No	Yes	Yes	Yes	Yes	5
Reuben et al. (2022)	No	Yes	Yes	No	Yes	Yes	Yes	Yes	5

The majority of the studies were of good quality, scoring 5 or above. Brenner et al. (2018) and (Hoch & Youssef, 2020) were the strongest-rated studies. Both used diagnoses from clinicians, the latter using electronic health records. In terms of the latter, it should be noted that there are sometimes inaccuracies in record keeping. Other studies used validated questionnaires and scales to measure PTSD symptoms or severity; however, the majority of these were self-report so were subject to possible self-report bias. Two papers, a primary and secondary analysis of the same data set, did not score well (score of 2) and scored low on analysis clarity and presentation of statistical results (Kupferstein, 2018, 2020).

With regards to selection bias, all of the studies recruited based on whether the participants had an autism diagnosis, with four studies having an additional criterion that participants had to be trauma exposed (Hoover & Romero, 2019; Lobregt-van Buuren et al., 2019; Rumball et al., 2020, 2021a). For the studies that did not stipulate trauma exposure, thus capturing a more representative autistic population, five attempted to prevent selection bias by not using the word ‘trauma’ in recruitment materials (Rumball et al., 2021b) or deliberately focusing recruitment advertisements around topics other than trauma, such as dissociation (Reuben et al., 2021, 2022) or ‘life events’ (Golan et al., 2022; Haruvi-Lamdan et al., 2020).

Five studies recruited exclusively from clinical services (Brenner et al., 2018; Hoover & Romero, 2019; Lobregt-van Buuren et al., 2019), two of which were limited to a specific centre: a community mental health provider in Midwest USA (Hoch & Youssef, 2020), and an ASD expert centre in Bordeaux, France (Paul et al., 2018). The rest of the studies used more than one mode of recruitment, including autism charities, clinics, activity centres, recruitment lists, social media, and online and off-line community adverts.

One group study recruited young boys only (Bitsika & Sharpley, 2021), the rest had mixed-sex samples. All studies that took place online had majority female samples, where those with in-person testing were majority male. The majority of the studies did not distinguish between sex and gender and use these terms interchangeably: only two studies by the same author considered both sex and gender identity and investigated PTSD in non-binary autistic people (Reuben et al., 2021, 2022).

Included group studies were generally biased towards verbal autistic participants that had average to above average IQ. Only one study stated that IQ level was not grounds for exclusion (Brenner et al., 2018); 42% of their sample had an IQ lower than 70 (the cut-off for ID). Six studies excluded (or did not include) those with ID (Bitsika & Sharpley, 2021; Golan et al., 2022; Haruvi-Lamdan et al., 2020; Lobregt-van Buuren et al., 2019; Paul et al., 2018; Rumball et al., 2020) and seven did not report if ID or IQ level was part of the exclusion criteria (Bitsika & Sharpley, 2021; Hoch & Youssef, 2020; Hoover & Romero, 2019; Kupferstein, 2018, 2020; Reuben et al., 2021, 2022; Rumball et al., 2021b). Hoover and Romero (2019) do not report IQ, but do state that specific verbal ability was not used as an inclusion criterion.

None of the included studies report co-production or co-design with autistic people. Only one study protocol was reviewed by people with lived-experience of mental health problems in the NIHR Feasibility and Acceptability Support Team for Researchers (FAST-R) (Rumball et al., 2020), and their suggestions were implemented into the study design.

## Discussion

This systematic review updated and expanded on the original review by Rumball (2019) which summarised studies published up until 2017. The present review highlights an increase in research interest in PTSD in autistic populations in the last 6 years, as illustrated by the number of included studies reporting PTSD symptoms in autistic individuals. Since Rumball’s (2019) review, there has been a general reconceptualization of PTSD away from the anxiety disorder classification and into a ‘trauma and stressor-related disorder’ category in the DSM-5. All but one (Hoch & Youssef, 2020) of the currently included studies reported use of PTSD symptom measures, in contrast to (Rumball, 2019) review, in which no studies did this. Our findings suggest that, when compared to their non-autistic peers, trauma-exposed autistic adults and children had more PTSD symptoms, and our estimates suggest that PTSD occurs at comparable rates in autistic individuals compared to the general population rates. We also include the first pilot study to investigate the efficacy of a PTSD treatment (EMDR therapy) in autistic people.

The literature reviewed suggests that autistic people are vulnerable to developing PTSD symptoms, potentially with higher severity than their non-autistic peers. First, the synthesis of case–control studies showed PTSD symptoms were higher in autistic adults and children when compared to non-autistic individuals and suggested certain PTSD symptom domains, such as some intrusion symptoms and negative cognitions, were higher in autistic groups (see Table 3). Higher PTSD symptoms could be impacted by the higher trauma exposure found in the autistic participants, as the case–control groups were not matched for levels of trauma exposure. Therefore, it remains untested if differences in PTSD symptom severity in the autistic people were due to increased vulnerability to PTSD or simply higher levels of trauma exposure in the autistic groups. There were also symptom domains where autistic groups showed no significant difference from non-autistic groups (Table 3). This bears clinical relevance as, for example, no significant difference in the presence of reflective rumination (Golan et al., 2022) suggests that reflective abilities are an equally meaningful resilience mechanism for autistic adults and may be a viable skill to teach or enhance during trauma-focused therapy.

Rumball and colleagues (Rumball et al. 2021a, b) have suggested that features of cognitive style in autistic people may predispose to or alter the development of PTSD symptoms. Cognitive models of PTSD suggest that cognitive processing during the traumatic event, as well as negative appraisals, disjointed memories, and maladaptive coping strategies, leads to the development and maintenance of PTSD symptoms in the general population (Ehlers & Clark, 2000). Thus, specific cognitive styles and sensory memory encoding, alongside predisposition for emotional dysregulation and sensory arousal, may impact the formation of trauma memories and present a risk pathway to traumatic sequelae to which autistic people may be especially prone. Included studies suggested that specific cognitive features that are known risk factors for PTSD in the general population may also augment PTSD symptomology in autistic adults. Observational group studies showed autistic adults had higher brooding rumination and poorer working and everyday memory than non-autistic groups, which significantly mediated the co-occurrence of autism and PTSD symptoms (Golan et al., 2022; Rumball et al., 2021b). Thought suppression was also associated with higher PTSD symptom score in autistic individuals (Rumball et al., 2021a). These findings are from modest-scale observational studies (sample size ranging from 34 to 59), so only provide initial estimates. However, they are echoed anecdotally by case studies that highlighted excessive rumination, poor recall, and being non-speaking as factors that impacted the presentation of PTSD in some autistic people (Carmassi et al., 2019; Fazel et al., 2020; Kildahl & Jørstad, 2022). Controlled studies recruiting trauma exposed autistic people with and without PTSD and non-autistic comparison groups are needed to understand how characteristics of autism interact with trauma experience and PTSD development. It has been suggested that certain cognitive tasks performed during or after a potential trauma may impact memory consolidation and thus reduce involuntary nature of subsequent intrusive thoughts (Holmes et al., 2009). There are autistic characteristics that may prove similarly protective or present alternative coping strategies that may currently be interpreted negatively in deficit-based frameworks of autism, such as self-regulating behaviours (e.g. stimming and visual-based special interests) (Ng-Cordell et al., 2022). To identify those most at risk and effectively manage post-traumatic stress in autistic people, we need to further understand specific cognitive strengths and vulnerabilities that autistic people have in relation to the development and maintenance of PTSD.

There has not, to date, been an epidemiological exploration of the prevalence of PTSD in autistic people in population-representative samples. In its absence, our estimated rates from the current literature suggest that autistic adults and children experience PTSD at, at least, similar rates to non-autistic population estimates. We collated autistic samples from existing literature using the results from both the present and Rumball’s (2019) review to calculate estimates of the percentage of autistic children and adults reporting PTSD diagnosis at any point in their lifetime (c. 5% and 16%, respectively) and rate of children (c. 14%) and adults (c. 44%) meeting PTSD symptom questionnaire cut-offs at the time of the studies. It is crucial to consider these results as a preliminary estimate, as the generalisability of these estimated rates is constrained to the populations that took part in the current available literature, which is not reflective of the diversity of people within the autistic community. These collated estimates, and the rates reported in individual studies, are comparable to those for non-autistic populations, which also vary significantly from study to study (Schein et al., 2021). In the UK, a large nationally representative birth cohort of 2232 twins at age 18 years showed that 4.4% had experienced PTSD in the past 12 months, and 7.8% had experienced PTSD at some point (Lewis et al., 2019). A systematic review synthesised 38 recent (2015–2019) studies on PTSD in US populations and found lifetime prevalence for adult civilians varied significantly and ranged from 3.4 to 26.9% (Schein et al., 2021). Rates of PTSD in studies using professional diagnosis were far lower than those using symptom questionnaire cut-offs (such as the PCL-5). In the current review, the two studies that included both PCL-5 scores and self-reported professional diagnosis for their participants also showed that the number of participants meeting PCL-5 cut-off for PTSD was approximately double that of those with a professional diagnosis (Reuben et al., 2022; Rumball et al., 2021a). This raises two concerns; first, autistic people with trauma-related symptoms are going unrecognised and untreated. Second, given the potential overlap in presentations of ASD and PTSD (Stavropoulos et al., 2018), existing assessment tools may be overly sensitive for this population; perhaps ‘picking up’ autistic traits instead of trauma symptoms.

Since Rumball’s (2019) review, there has been little progress in validating existing symptom scales to ensure they are fit for purpose in autistic people. In a recent Delphi study, less than 20% of experts endorsed accuracy of trauma measures for use in autistic young people (Kerns et al., 2022). All of the studies included here that reported on PTSD in adults used the PCL-5, a highly validated 20-item self-report checklist based on the DSM-5 criteria, with strong internal consistency, test-rest reliability, and convergent and discriminant validity (Blevins et al., 2015; Bovin et al., 2016). However, the PCL-5 has never been validated in autistic individuals. Although preliminary psychometric analyses of the interactive trauma scale, which was piloted with 20 autistic children, suggest convergent validity with the child self-report and parent-report forms of the UCLA PTSD-RI (Hoover & Romero, 2019), there are no validated, clinically available PTSD scales for use with autistic people. Furthermore, the symptoms investigated in the included studies were confined to those used to diagnose PTSD in neurotypical people. Future research is needed to investigate if there are autism-specific presentations of PTSD that fall outside of the established symptomology, as well as ensuring there are validated diagnostic tools that can assess and discriminate PTSD symptoms from characteristics of autism.

Clinicians may be overlooking trauma-related symptoms in response to events experienced by autistic people as traumas, which otherwise fall outside of traditional definitions. Increasingly, literature supports the importance of subjective experience of traumatic events in developing PTSD and other types of psychopathologies (Brewin et al., 2019; Danese & Widom, 2020) and the importance of exploring the role of autistic perception and experience on what is found to be traumatising (Kerns et al., 2015). PTSD symptoms can and do arise from events that do not qualify as traumatic according to the DSM-5 definition, and several included studies by Rumball and colleagues explored this in autistic adults and found no difference in risk of PTSD associated with DSM-5 and non-DSM-5 qualifying traumas (Rumball et al., 2020). It has been hypothesised that rates of trauma exposure could be exacerbated by autistic people experiencing a wider range of events as traumatic, although when compared to non-autistic adults, the frequency of these DSM and non-DSM traumas did not differ between the groups (Rumball et al., 2021b). Complex PTSD following repeated exposure to circumstances experienced as traumatic has yet to be empirically explored in autistic children or adults, as revealed by the present review. Given the reported impact of more consistent day-to-day difficulties (e.g. stigma) on the mental health of autistic people (Ghanouni & Quirke, 2023), this warrants investigation. There are also experiences that are specific to autistic people that may not be considered when trauma is conceptualised in neurotypical populations. Applied behavioural analysis (ABA), an autism intervention, and its relationship to PTSD symptoms were explored by Kupferstein (2018, 2020) in two papers from the same study. Some autism advocates have raised concerns about the safety of ABA (Wilkenfeld & McCarthy, 2020). In this study, it was suggested that the therapy that set out to help autistic children learn adaptive behaviours, as designed and delivered by neurotypical clinicians, resulted in post-traumatic stress and adverse effects. However, caution must be taken when drawing conclusions about ABA’s relationship to PTSD from this study, due to significant methodological flaws (e.g. poor analysis clarity), as discussed by Leaf et al. (2018). More generally, adopting a wide definition of trauma and focusing on individuals’ perception of, and PTSD-like responses to, experiences, would appear to be most fruitful for the study of PTSD symptoms in autistic people.

In terms of treatment of PTSD (symptoms), this review highlights that there is still a scarcity of research on how best to approach PTSD treatment in autistic people. To date, only one evidence-based treatment for PTSD—EMDR—has been empirically tested and shown to significantly reduce PTSD symptom severity in autistic individuals (Lobregt-van Buuren et al., 2019), and there are no investigations into how being autistic may impact the success of PTSD treatments. EMDR has been suggested to be adaptable for autistic clients (Fisher et al., 2022), and its successful use as an add-on therapy in a relatively small sample indicates its therapeutic promise (Lobregt-van Buuren et al., 2019). However, well-powered randomised controlled trials are still needed to assess if the quality and effectiveness of EMDR surpasses TAU in autistic people diagnosed with PTSD. Likewise, trauma-focused CBT (TF-CBT) is the first-line treatment for PTSD in the general population and shows high efficacy across ages (Kar, 2011) but remains to be systematically investigated in relation to PTSD in autistic individuals. Autism-specific adaptations of TF-CBT have been theoretically proposed in areas of emotion regulation, graduated exposure, cognitive restructuring, and psychoeducation (Stack & Lucyshyn, 2019), and randomised controlled trials have shown efficacy of adapting CBT to treat depression in autistic adults (Russell et al., 2019a) and anxiety in autistic adolescents (Storch et al., 2015). For psychotherapies, more generally, the person-environment fit is an essential consideration and specific adaptations should be considered when a patient is autistic (Brook, 2023; Mazurek et al., 2023). Anecdotal reports highlight the importance of careful consideration of the individual needs of an autistic person suffering from PTSD for successful use of psychological therapies, including narrative exposure therapy (NET) (Fazel et al., 2020) and adapted CBT (Carmassi et al., 2019), and use of trauma-informed care to manage symptoms (Kildahl & Jørstad, 2022). There is a clinical need to empirically assess the efficacy of adaptations of evidence-based PTSD treatment in autistic populations, particularly first-line treatment employing TF-CBT.

All but one of the studies in this review required active recruitment of autistic people, affecting the generalisability of these findings as these studies were biased towards autistic people with an official diagnosis who had the ability and motivation to volunteer for these studies. As in Rumball (2019) review, autistic people with co-occurring ID continue to be under-represented in the studies identified in this review and in the autism research field more widely (Jack & Pelphrey, 2017; Russell et al., 2019a, b). Low IQ has been associated with PTSD development in neurotypical populations (Breslau et al., 2006). Given that a population study showed around a third of autistic adults also report ID (Rydzewska et al., 2018), co-occurring ID may increase risk of PTSD development and make identification of PTSD more difficult in some autistic people (Borghus et al., 2018; Mevissen & de Jongh, 2010). ID may also bring specific barriers to an autistic person’s ability to self-advocate and access care for PTSD. Therefore, it is important to understand how trauma symptoms manifest in this population. Autistic individuals with ID are more likely to have specific care needs (Cooper et al., 2006; Hewitt et al., 2017), and reliance on disability services may make them vulnerable to new, or maintain existing, traumatising situations. There is a push towards promoting a trauma-informed care model in these services (Rich et al., 2021). This is exemplified by the case study describing PTSD symptoms of an autistic man traumatised by scalding from being washed by his carer (Kildahl & Jørstad, 2022). In this case, a trauma-informed care model was effective in managing PTSD symptoms after an exposure-based intervention had made the individual’s avoidance symptoms worse. This highlights the importance of considering the individual’s perception of treatment and thus their subsequent feelings of safety. In a qualitative investigation of clinicians’ perspectives on the ways PTSD may be overlooked in autistic patients with ID, Kildhahl and colleagues (2020) highlighted that for these patients, avoidance symptoms appeared less specific to stimuli associated with the trauma, and avoidance was less planned (more reactionary). The clinicians highlighted that this more generalised response may make PTSD symptoms less recognisable and patients more vulnerable to upsetting (triggering) stimuli. As the majority of included studies excluded participants with ID, there is a fundamental gap in the literature and a pressing need for studies taking a more inclusive approach to empirically investigate trauma-related symptoms in autistic people with co-occurring ID.

On the issue of recruiting gender-diverse samples; some progress has been made with the majority of the studies including autistic women and girls, who have, historically, been under-represented in autism research. This is particularly important to the study of PTSD, which in the general population is more common amongst women (Olff, 2017). Additionally, research shows that transgender people are at the high risk of PTSD (Marchi et al., 2023; Reisner et al., 2016). A large population study showed higher numbers of transgender and gender diverse people are autistic when compared to cisgender people (Warrier et al., 2020). Only two included studies by the same researchers explicitly asked about and compared people on a basis of gender identity (Reuben et al., 2021, 2022). Future work should not only explore the distinction between how sex and gender identity impact PTSD risk and presentation but also ensure that this critical information is not erased by binary demographic data. Additionally, efforts should be made to specifically recruit autistic individuals from marginalised populations to explore intersectionality of PTSD risk.

### Limitations

This review systematically identified literature pertaining to the assessment, prevalence, and treatment of PTSD in autistic people, since the termination of Rumball’s (2019) search in 2017. This systematic review is limited by its inclusion criteria, which only considered studies using standardised PTSD scales and diagnoses, and studies pertaining to those with an official autism diagnosis. This will exclude those who self-identify as autistic and introduce a bias towards studies with those who have the means to access an autism diagnosis. Additionally, given the hypothesis that diagnostic overshadowing may lead to missed PTSD diagnosis in autistic people, excluded papers investigating the impact of adverse childhood experiences and bullying on autistic people’s mental health, as well as studies demonstrating the association between ASD traits and trauma exposure or PTSD risk in adults (Haruvi-Lamdan et al., 2019; Stewart et al., 2022), could provide valuable information on the unrecognised trauma-related symptoms experienced by autistic individuals. Qualitative studies involving medical professionals and community service providers were also excluded, yet provide important insights into the assessment, diagnosis, and treatment of PTSD in autistic individuals. Our quality assessment rated studies that used clinical diagnoses of PTSD as the highest quality papers, and some studies were penalised for using self-report questionnaire cut-offs, which were not deemed objective methods of measurement by quality assessment tools. This was, in part, due to these PTSD questionnaires being subject to self-report bias. However, self-report measures can also be a valuable mode of mental health assessment, allowing insight into the perspectives of the patient and avoiding possible clinician bias. This may be of particular importance for autistic people, for whom interacting with mental healthcare professionals may be particularly difficult (Au-Yeung et al., 2019; Brede et al., 2022).

### Conclusion

Untreated, PTSD has a profound impact on an individual’s psychological wellbeing. This systematic review highlights that, when compared to their non-autistic peers, trauma-exposed autistic individuals display more severe PTSD symptoms, with at least comparable rates of occurrence. Despite there being promising research exploring PTSD in autistic adults and children since Rumball’s (2019) review, it is crucial that future research validates PTSD symptom assessment tools, explores unique challenges and manifestations of trauma-related symptoms in autistic individuals, and involves the autistic community to understand research priorities and views around experiencing PTSD as an autistic person. This will ultimately lead to effective ways to diagnose and address PTSD in autistic children and adults.

## References

[CR1] American Psychiatric Association. (2013). *Diagnostic and Statistical Manual of Mental Disorders (DSM-5®)* (5th ed.). American Psychiatric Pub.

[CR2] Au-Yeung, S. K., Bradley, L., Robertson, A. E., Shaw, R., Baron-Cohen, S., & Cassidy, S. (2019). Experience of mental health diagnosis and perceived misdiagnosis in autistic, possibly autistic and non-autistic adults. *Autism,**23*(6), 1508–1518. 10.1177/1362361318818167 30547677 10.1177/1362361318818167

[CR3] *Bitsika, V., & Sharpley, C. F. (2021). Direct and inverse correlates of post-traumatic stress disorder among school-age autistic boys. *International Journal of Environmental Research and Public Health.,**18*(10), 5285. 10.3390/ijerph18105285 34065676 10.3390/ijerph18105285 PMC8155909

[CR4] Blevins, C. A., Weathers, F. W., Davis, M. T., Witte, T. K., & Domino, J. L. (2015). The Posttraumatic Stress Disorder Checklist for DSM-5 (PCL-5): Development and initial psychometric evaluation. *Journal of Traumatic Stress,**28*(6), 489–498. 10.1002/jts.22059 26606250 10.1002/jts.22059

[CR5] Borghus, A., Dokkedahl, S., & Elklit, A. (2018). Pilot study: Undetected post-traumatic stress disorder symptoms among intellectually disabled. *International Journal of Developmental Disabilities,**66*(1), 36–45. 10.1080/20473869.2018.1475539 34141365 10.1080/20473869.2018.1475539 PMC8115620

[CR6] Bovin, M. J., Marx, B. P., Weathers, F. W., Gallagher, M. W., Rodriguez, P., Schnurr, P. P., & Keane, T. M. (2016). Psychometric properties of the PTSD checklist for diagnostic and statistical manual of mental disorders-fifth edition (PCL-5) in veterans. *Psychological Assessment,**28*(11), 1379–1391. 10.1037/pas0000254 26653052 10.1037/pas0000254

[CR7] Brede, J., Cage, E., Trott, J., Palmer, L., Smith, A., Serpell, L., Mandy, W., & Russell, A. (2022). “We Have to Try to Find a Way, a Clinical Bridge” - autistic adults’ experience of accessing and receiving support for mental health difficulties: A systematic review and thematic meta-synthesis. *Clinical Psychology Review,**93*, 102131. 10.1016/j.cpr.2022.102131 35180632 10.1016/j.cpr.2022.102131

[CR8] *Brenner, J., Pan, Z., Mazefsky, C., et al. (2018). Behavioral symptoms of reported abuse in children and adolescents with autism spectrum disorder in inpatient settings. *Journal of Autism and Developmental Disorders,**48*, 3727–3735. 10.1007/s10803-017-3183-4 28593599 10.1007/s10803-017-3183-4

[CR9] Breslau, N., Lucia, V. C., & Alvarado, G. F. (2006). Intelligence and other predisposing factors in exposure to trauma and posttraumatic stress disorder: A follow-up study at age 17 years. *Archives of General Psychiatry,**63*(11), 1238–1245. 10.1001/archpsyc.63.11.1238 17088504 10.1001/archpsyc.63.11.1238

[CR10] Brewin, C. R., Rumball, F., & Happé, F. (2019). Neglected causes of post-traumatic stress disorder. *BMJ,**365*, l2372. 10.1136/bmj.l2372 31182425 10.1136/bmj.l2372

[CR11] Brook, Y. (2023). How talking therapists experience working with adult clients who have autism. *Counselling and Psychotherapy Research,**23*(1), 235–246. 10.1002/capr.12591

[CR13] *Carmassi, C., Bertelloni, C. A., Salarpi, G., Diadema, E., Avella, M. T., Dell’Oste, V., & Dell’Osso, L. (2019). Is there a major role for undetected autism spectrum disorder with childhood trauma in a patient with a diagnosis of bipolar disorder, self-injuring, and multiple comorbidities? *Case Reports in Psychiatry,**2019*, 4703795. 10.1155/2019/4703795 31249714 10.1155/2019/4703795 PMC6556326

[CR14] Cooper, S.-A., Morrison, J., Melville, C., Finlayson, J., Allan, L., Martin, G., & Robinson, N. (2006). Improving the health of people with intellectual disabilities: outcomes of a health screening programme after 1 year. *Journal of Intellectual Disability Research,**50*, 667–677. 10.1111/j.1365-2788.2006.00824.x 16901294 10.1111/j.1365-2788.2006.00824.x

[CR15] Danese, A., & Widom, C. S. (2020). Objective and subjective experiences of child maltreatment and their relationships with psychopathology. *Nature Human Behaviour,**4*(8), 811–818. 10.1038/s41562-020-0880-3 32424258 10.1038/s41562-020-0880-3

[CR12] de Bruin, E. I., Ferdinand, R. F., Meester, S., de Nijs, P. F., & Verheij, F. (2007). High rates of psychiatric comorbidity in PDD-NOS. *Journal of Autism and Developmental Disorders,**37*(5), 877–886. 17031447 10.1007/s10803-006-0215-x

[CR16] Ehlers, A., & Clark, D. M. (2000). A cognitive model of posttraumatic stress disorder. *Behaviour Research and Therapy,**38*(4), 319–345. 10761279 10.1016/s0005-7967(99)00123-0

[CR17] *Fazel, M., Stratford, H. J., Rowsell, E., Chan, C., Griffiths, H., & Robjant, K. (2020). Five applications of narrative exposure therapy for children and adolescents presenting with post-traumatic stress disorders. *Frontiers in Psychiatry,**11*, 19. 10.3389/fpsyt.2020.00019 32140112 10.3389/fpsyt.2020.00019 PMC7043101

[CR18] Fisher, N., van Diest, C., Leoni, M., & Spain, D. (2022). Using EMDR with autistic individuals: A Delphi survey with EMDR therapists. *Autism*, 13623613221080254. 10.1177/13623613221080254 10.1177/13623613221080254 PMC9806468 35384753

[CR19] Ghanouni, P., & Quirke, S. (2023). Resilience and coping strategies in adults with autism spectrum disorder. *Journal of Autism and Developmental Disorders,**53*, 456–467. 10.1007/s10803-022-05436-y 35079928 10.1007/s10803-022-05436-y PMC8788904

[CR20] *Golan, O., Haruvi-Lamdan, N., Laor, N., & Horesh, D. (2022). The comorbidity between autism spectrum disorder and post-traumatic stress disorder is mediated by brooding rumination. *Autism,**26*(2), 538–544. 10.1177/13623613211035240 34318687 10.1177/13623613211035240

[CR21] Han, E., Scior, K., Avramides, K., & Crane, L. (2022). A systematic review on autistic people’s experiences of stigma and coping strategies. *Autism Research,**15*(1), 12–26. 10.1002/aur.2652 34881514 10.1002/aur.2652

[CR22] Haruvi-Lamdan, N., Horesh, D., & Golan, O. (2018). PTSD and autism spectrum disorder: Co-morbidity, gaps in research, and potential shared mechanisms. *Psychological Trauma: Theory, Research, Practice, and Policy,**10*(3), 290. 10.1037/tra0000298 28726442 10.1037/tra0000298

[CR23] *Haruvi-Lamdan, N., Horesh, D., Zohar, S., Kraus, M., & Golan, O. (2020). Autism spectrum disorder and post-traumatic stress disorder: An unexplored co-occurrence of conditions. *Autism,**24*(4), 884–898. 10.1177/1362361320912143 32245333 10.1177/1362361320912143

[CR24] Haruvi-Lamdan, N., Lebendiger, S., Golan, O., & Horesh, D. (2019). Are PTSD and autistic traits related? An examination among typically developing Israeli adults. *Comprehensive Psychiatry,**89*, 22–27. 10.1016/j.comppsych.2018.11.004 30579126 10.1016/j.comppsych.2018.11.004

[CR25] Hewitt, A. S., Stancliffe, R. J., Hall-Lande, J., Nord, D., Pettingell, S. L., Hamre, K., & Hallas-Muchow, L. (2017). Characteristics of adults with autism spectrum disorder who use residential services and supports through adult developmental disability services in the United States. *Research in Autism Spectrum Disorders,**34*, 1–9. 10.1016/j.rasd.2016.11.007

[CR26] *Hoch, J. D., & Youssef, A. M. (2020). Predictors of trauma exposure and trauma diagnoses for children with autism and developmental disorders served in a community mental health clinic. *Journal of Autism and Developmental Disorders,**50*(2), 634–649. 10.1007/s10803-019-04331-3 31838644 10.1007/s10803-019-04331-3 PMC6994449

[CR27] Hofvander, B., Delorme, R., Chaste, P., Nydén, A., Wentz, E., Ståhlberg, O., et al. (2009). Psychiatric and psychosocial problems in adults with normal-intelligence autism spectrum disorders. *BMC Psychiatry,**9*(1), 35. 19515234 10.1186/1471-244X-9-35 PMC2705351

[CR29] Hollocks, M. J., Pickles, A., Howlin, P., & Simonoff, E. (2016). Dual cognitive and biological correlates of anxiety in autism spectrum disorders. *Journal of Autism and Developmental Disorders,**46*(10), 3295–3307. 27465243 10.1007/s10803-016-2878-2

[CR28] Holmes, E. A., James, E. L., Coode-Bate, T., & Deeprose, C. (2009). Can playing the computer game “Tetris” reduce the build-up of flashbacks for trauma? A proposal from cognitive science. *PLoS ONE,**4*(1), e4153. 10.1371/journal.pone.0004153 19127289 10.1371/journal.pone.0004153 PMC2607539

[CR30] Hoover, D. W. (2020). Trauma in children with neurodevelopmental disorders: Autism, intellectual disability, and attention-deficit/hyperactivity disorder. In G. Spalletta, D. Janiri, F. Piras, & G. Sani (Eds.), *Childhood trauma in mental disorders: A comprehensive approach* (pp. 367–383). Springer International Publishing. 10.1007/978-3-030-49414-8_17

[CR31] Hoover, D. W., & Kaufman, J. (2018). Adverse childhood experiences in children with autism spectrum disorder. *Current Opinion in Psychiatry,**31*(2), 128–132. 10.1097/yco.0000000000000390 29206686 10.1097/YCO.0000000000000390 PMC6082373

[CR32] *Hoover, D. W., & Romero, E. M. (2019). The interactive trauma scale: A web-based measure for children with autism. *Journal of Autism and Developmental Disorders,**49*(4), 1686–1692. 10.1007/s10803-018-03864-3 30604349 10.1007/s10803-018-03864-3

[CR33] Jack, A., & Pelphrey, K. A. (2017). Annual research review: Understudied populations within the autism spectrum – current trends and future directions in neuroimaging research. *J Child Psychol Psychiatr,**58*, 411–435. 10.1111/jcpp.12687 10.1111/jcpp.12687 PMC5367938 28102566

[CR34] Kar, N. (2011). Cognitive behavioral therapy for the treatment of post-traumatic stress disorder: A review. *Neuropsychiatric Disease and Treatment,**7*, 167–181. 10.2147/ndt.S10389 21552319 10.2147/NDT.S10389 PMC3083990

[CR35] Kerns, C. M., Berkowitz, S. J., Moskowitz, L. J., Drahota, A., Lerner, M. D., & Newschaffer, C. J. (2020a). Screening and treatment of trauma-related symptoms in youth with autism spectrum disorder among community providers in the United States. *Autism,**24*(2), 515–525. 10.1177/1362361319847908 31200605 10.1177/1362361319847908 PMC6911025

[CR36] Kerns, C. M., Newschaffer, C. J., & Berkowitz, S. J. (2015). Traumatic childhood events and autism spectrum disorder. *Journal of Autism and Developmental Disorders,**45*(11), 3475–3486. 10.1007/s10803-015-2392-y 25711547 10.1007/s10803-015-2392-y

[CR37] Kerns, C. M., Rast, J. E., & Shattuck, P. T. (2020b). Prevalence and correlates of caregiver-reported mental health conditions in youth with autism spectrum disorder in the United States. *The Journal of Clinical Psychiatry*, *82*(1). 10.4088/JCP.20m13242 10.4088/JCP.20m13242 33356021

[CR38] Kerns, C. M., Robins, D. L., Shattuck, P. T., Newschaffer, C. J., & Berkowitz, S. J. (2022). Expert consensus regarding indicators of a traumatic reaction in autistic youth: A Delphi survey. *Journal of Child Psychology and Psychiatry*. 10.1111/jcpp.13666 35817758 10.1111/jcpp.13666 PMC10368297

[CR39] Kildahl, A. N., Helverschou, S. B., Bakken, T. L., & Oddli, H. W. (2020). “Driven and tense, stressed out and anxious”: Clinicians’ perceptions of post-traumatic stress disorder symptom expressions in adults with autism and intellectual disability [Article]. *Journal of Mental Health Research in Intellectual Disabilities,**13*(3), 201–230. 10.1080/19315864.2020.1760972

[CR40] *Kildahl, A. N., & Jørstad, I. (2022). Post-traumatic stress disorder symptom manifestations in an autistic man with severe intellectual disability following coercion and scalding. *Journal of Intellectual & Developmental Disability,**47*(2), 190–194. 10.3109/13668250.2021.1995930 39818586 10.3109/13668250.2021.1995930

[CR41] *Kupferstein, H. (2018). Evidence of increased PTSD symptoms in autistics exposed to applied behavior analysis [Article]. *Advances in Autism,**4*(1), 19–29. 10.1108/AIA-08-2017-0016

[CR42] *Kupferstein, H. (2020). Why caregivers discontinue applied behavior analysis (ABA) and choose communication-based autism interventions [Article]. *Advances in Autism,**6*(1), 72–80. 10.1108/AIA-02-2019-0004

[CR43] Leaf, J. B., Ross, R. K., Cihon, J. H., & Weiss, M. J. (2018). Evaluating Kupferstein’s claims of the relationship of behavioral intervention to PTSS for individuals with autism. *Advances in Autism,**4*(3), 122–129. 10.1108/AIA-02-2018-0007

[CR44] Lewis, S. J., Arseneault, L., Caspi, A., Fisher, H. L., Matthews, T., Moffitt, T. E., Odgers, C. L., Stahl, D., Teng, J. Y., & Danese, A. (2019). The epidemiology of trauma and post-traumatic stress disorder in a representative cohort of young people in England and Wales. *Lancet Psychiatry,**6*(3), 247–256. 10.1016/s2215-0366(19)30031-8 30798897 10.1016/S2215-0366(19)30031-8 PMC6384243

[CR45] Lobregt-van Buuren, E., Hoekert, M., & Sizoo, B. (2021). Autism, adverse events, and trauma. In A. M. Grabrucker (Ed.), *Autism Spectrum Disorders*. Exon Publications. 34495617

[CR46] *Lobregt-van Buuren, E., Sizoo, B., Mevissen, L., & de Jongh, A. (2019). Eye movement desensitization and reprocessing (EMDR) therapy as a feasible and potential effective treatment for adults with autism spectrum disorder (ASD) and a history of adverse events. *Journal of Autism and Developmental Disorders,**49*(1), 151–164. 30047096 10.1007/s10803-018-3687-6

[CR48] Marchi, M., Travascio, A., Uberti, D., De Micheli, E., Grenzi, P., Arcolin, E., Pingani, L., Ferrari, S., & Galeazzi, G. M. (2023). Post-traumatic stress disorder among LGBTQ people: A systematic review and meta-analysis. *Epidemiology and Psychiatric Sciences,**32*, e44. 10.1017/S2045796023000586 37431310 10.1017/S2045796023000586 PMC10387489

[CR49] Mazurek, M. O., Pappagianopoulos, J., Brunt, S., Sadikova, E., Nevill, R., Menezes, M., & Harkins, C. (2023). A mixed methods study of autistic adults’ mental health therapy experiences. *Clinical Psychology & Psychotherapy,**30*(4), 767–779. 10.1002/cpp.2835 36708045 10.1002/cpp.2835 PMC10372197

[CR47] Mcconachie, H., McLaughlin, E., Grahame, V., Taylor, H., Honey, E., Tavernor, L., et al. (2014). Group therapy for anxiety in children with autism spectrum disorder. *Autism,**18*(6), 723–732. 24101715 10.1177/1362361313488839

[CR50] Mehtar, M., & Mukaddes, N. M. (2011). Posttraumatic stress disorder in individuals with diagnosis of autistic spectrum disorders. *Research in Autism Spectrum Disorders,**5*(1), 539–546.

[CR51] Mevissen, L., & de Jongh, A. (2010). PTSD and its treatment in people with intellectual disabilities: A review of the literature. *Clinical Psychology Review,**30*(3), 308–316. 10.1016/j.cpr.2009.12.005 20056303 10.1016/j.cpr.2009.12.005

[CR52] Ng-Cordell, E., Rai, A., Peracha, H., Garfield, T., Lankenau, S. E., Robins, D. L., Berkowitz, S. J., Newschaffer, C., & Kerns, C. M. (2022). A qualitative study of self and caregiver perspectives on how autistic individuals cope with trauma. *Frontiers in Psychiatry,**13*, 825008. 10.3389/fpsyt.2022.825008 35911211 10.3389/fpsyt.2022.825008 PMC9329569

[CR53] Olff, M. (2017). Sex and gender differences in post-traumatic stress disorder: An update. *European Journal of Psychotraumatology,**8*(sup4), 1351204. 10.1080/20008198.2017.1351204

[CR54] Page, M. J., Moher, D., Bossuyt, P. M., Boutron, I., Hoffmann, T. C., Mulrow, C. D., Shamseer, L., Tetzlaff, J. M., Akl, E. A., Brennan, S. E., Chou, R., Glanville, J., Grimshaw, J. M., Hróbjartsson, A., Lalu, M. M., Li, T., Loder, E. W., Mayo-Wilson, E., McDonald, S., … McKenzie, J. E. (2021). PRISMA 2020 explanation and elaboration: Updated guidance and exemplars for reporting systematic reviews. *BMJ*, *372*, n160. 10.1136/bmj.n160 10.1136/bmj.n160 PMC8005925 33781993

[CR55] *Paul, A., Gallot, C., Lelouche, C., Bouvard, M. P., & Amestoy, A. (2018). Victimisation in a French population of children and youths with autism spectrum disorder: A case control study. *Child and Adolescent Psychiatry and Mental Health,**12*, 48. 10.1186/s13034-018-0256-x 30524501 10.1186/s13034-018-0256-x PMC6276214

[CR56] Peterson, J., Earl, R., Fox, E., Ma, R., Haidar, G., Pepper, M., Berliner, L., Wallace, A., & Bernier, R. (2019). Trauma and autism spectrum disorder: Review, proposed treatment adaptations and future directions. *Journal of Child and Adolescent Trauma,**12*(4), 529–547. 10.1007/s40653-019-00253-5 31819782 10.1007/s40653-019-00253-5 PMC6901292

[CR57] Reinvall, O., Moisio, A. L., Lahti-Nuuttila, P., Voutilainen, A., Laasonen, M., & Kujala, T. (2016). Psychiatric symptoms in children and adolescents with higher functioning autism spectrum disorders on the Development and Well-Being Assessment. *Research in Autism Spectrum Disorders,**25*, 47–57.

[CR58] Reisner, S. L., White Hughto, J. M., Gamarel, K. E., Keuroghlian, A. S., Mizock, L., & Pachankis, J. (2016). Discriminatory experiences associated with posttraumatic stress disorder symptoms among transgender adults. *Journal of Counseling Psychology,**63*(5), 509–519. 10.1037/cou0000143 26866637 10.1037/cou0000143 PMC4981566

[CR59] *Reuben, K. E., Self-Brown, S., & Vinoski Thomas, E. (2022). PTSD in autistic adults: Correlates of meeting DSM-5 criteria and predictors of professional diagnosis. *Psychological Trauma*. 10.1037/tra0001365 36174160 10.1037/tra0001365

[CR60] *Reuben, K. E., Stanzione, C. M., & Singleton, J. L. (2021). Interpersonal trauma and posttraumatic stress in autistic adults. *Autism in Adulthood,**3*(3), 247–256. 10.1089/aut.2020.0073 36605371 10.1089/aut.2020.0073 PMC8992908

[CR61] Rich, A. J., DiGregorio, N., & Strassle, C. (2021). Trauma-informed care in the context of intellectual and developmental disability services: Perceptions of service providers. *Journal of Intellectual Disabilities,**25*(4), 603–618. 10.1177/1744629520918086 32319343 10.1177/1744629520918086

[CR62] Rumball, F. (2019). A systematic review of the assessment and treatment of posttraumatic stress disorder in individuals with autism spectrum disorders. *Review Journal of Autism and Developmental Disorders,**6*(3), 294–324. 10.1007/s40489-018-0133-9

[CR63] *Rumball, F., Antal, K., Happé, F., & Grey, N. (2021a). Co-occurring mental health symptoms and cognitive processes in trauma-exposed ASD adults. *Research in Developmental Disabilities,**110*, 103836. 10.1016/j.ridd.2020.103836 33453693 10.1016/j.ridd.2020.103836

[CR64] *Rumball, F., Brook, L., Happé, F., & Karl, A. (2021b). Heightened risk of posttraumatic stress disorder in adults with autism spectrum disorder: The role of cumulative trauma and memory deficits. *Research in Developmental Disabilities,**110*, 103848. 10.1016/j.ridd.2020.103848 33454451 10.1016/j.ridd.2020.103848

[CR65] *Rumball, F., Happé, F., & Grey, N. (2020). Experience of trauma and PTSD symptoms in autistic adults: Risk of PTSD development following DSM-5 and non-DSM-5 traumatic life events. *Autism Research,**13*(12), 2122–2132. 10.1002/aur.2306 32319731 10.1002/aur.2306

[CR66] Russell, A., Gaunt, D. M., Cooper, K., Barton, S., Horwood, J., Kessler, D., Metcalfe, C., Ensum, I., Ingham, B., Parr, J. R., Rai, D., & Wiles, N. (2019a). The feasibility of low-intensity psychological therapy for depression co-occurring with autism in adults: The Autism Depression Trial (ADEPT) – A pilot randomised controlled trial. *Autism,**24*(6), 1360–1372. 10.1177/1362361319889272 31782656 10.1177/1362361319889272 PMC8645299

[CR67] Russell, G., Mandy, W., Elliott, D., White, R., Pittwood, T., & Ford, T. (2019b). Selection bias on intellectual ability in autism research: A cross-sectional review and meta-analysis. *Molecular Autism,**10*, 9. 10.1186/s13229-019-0260-x 30867896 10.1186/s13229-019-0260-x PMC6397505

[CR68] Rydzewska, E., Hughes-McCormack, L. A., Gillberg, C., Henderson, A., MacIntyre, C., Rintoul, J., & Cooper, S.-A. (2018). Prevalence of long-term health conditions in adults with autism: Observational study of a whole country population. *British Medical Journal Open,**8*(8), e023945. 10.1136/bmjopen-2018-023945 10.1136/bmjopen-2018-023945 PMC6120653 30173164

[CR69] Schein, J., Houle, C., Urganus, A., Cloutier, M., Patterson-Lomba, O., Wang, Y., King, S., Levinson, W., Guérin, A., Lefebvre, P., & Davis, L. L. (2021). Prevalence of post-traumatic stress disorder in the United States: A systematic literature review. *Current Medical Research and Opinion,**37*(12), 2151–2161. 10.1080/03007995.2021.1978417 34498953 10.1080/03007995.2021.1978417

[CR70] Smith, P., Dalgleish, T., & Meiser-Stedman, R. (2019). Practitioner review: Posttraumatic stress disorder and its treatment in children and adolescents. *Journal of Child Psychology and Psychiatry,**60*(5), 500–515. 10.1111/jcpp.12983 30350312 10.1111/jcpp.12983 PMC6711754

[CR71] Stack, A., & Lucyshyn, J. (2019). Autism spectrum disorder and the experience of traumatic events: Review of the current literature to inform modifications to a treatment model for children with autism. *Journal of Autism and Developmental Disorders,**49*(4), 1613–1625. 10.1007/s10803-018-3854-9 30539370 10.1007/s10803-018-3854-9

[CR72] Stavropoulos, K. K., Bolourian, Y., & Blacher, J. (2018). Differential diagnosis of autism spectrum disorder and post traumatic stress disorder: Two clinical cases. *Journal of Clinical Medicine,**7*(4), 71. 10.3390/jcm7040071 29642485 10.3390/jcm7040071 PMC5920445

[CR73] Stewart, G. R., Corbett, A., Ballard, C., et al. (2022) Traumatic life experiences and post-traumatic stress symptomsin middle-aged and older adults with and without autistic traits. *International Journal GeriatricPsychiatry*. 1–10. 10.1002/gps.5669 10.1002/gps.5669 34994472

[CR74] Storch, E. A., Lewin, A. B., Collier, A. B., Arnold, E., De Nadai, A. S., Dane, B. F., Nadeau, J. M., Mutch, P. J., & Murphy, T. K. (2015). A randomized controlled trial of cognitive-behavioral therapy versus treatment as usual for adolescents with autism spectrum disorders and comorbid anxiety. *Depression and Anxiety,**32*(3), 174–181. 10.1002/da.22332 10.1002/da.22332 PMC4346416 25424398

[CR75] Storch, E. A., Sulkowski, M. L., Nadeau, J., Lewin, A. B., Arnold, E. B., Mutch, P. J., et al. (2013). The phenomenology and clinical correlates of suicidal thoughts and behaviors in youth with autism spectrum disorders. *Journal of Autism and Developmental Disorders,**43*(10), 2450–2459. 23446993 10.1007/s10803-013-1795-x PMC3808993

[CR76] Taylor, J. L., & Gotham, K. O. (2016). Cumulative life events, traumatic experiences, and psychiatric symptomatology in transition-aged youth with autism spectrum disorder. *Journal of Neurodevelopmental Disorders,**8*(1), 28. 27468315 10.1186/s11689-016-9160-y PMC4962443

[CR77] The Joanna Briggs Institute. (2016). Checklist for case reports. http://joannabriggs.org/research/critical-appraisal-tools.html. Accessed Jan 2023.

[CR78] Turnock, A., Langley, K., & Jones, C. R. G. (2022). Understanding Stigma in Autism: A Narrative Reviewand Theoretical Model. *Autism in Adulthood: Challenges and Management,**4*(1), 76–91. 10.1089/aut.2021.0005 10.1089/aut.2021.0005 PMC8992913 36605561

[CR79] Warrier, V., Greenberg, D. M., Weir, E., Buckingham, C., Smith, P., Lai, M.-C., Allison, C., & Baron-Cohen, S. (2020). Elevated rates of autism, other neurodevelopmental and psychiatric diagnoses, and autistic traits in transgender and gender-diverse individuals. *Nature Communications,**11*(1), 3959. 10.1038/s41467-020-17794-1 32770077 10.1038/s41467-020-17794-1 PMC7415151

[CR80] Wells, G. A., Shea, B., O’Connell, D., Peterson, J., Welch, V., Losos, M., & Tugwell, P. (2012). The Newcastle-Ottawa Scale (NOS) for assessing the quality if nonrandomized studies in meta-analyses, 2012. Available from: https://www.ohri.ca/programs/clinical_epidemiology/oxford.asp

[CR82] White, S. W., Schry, A. R., & Maddox, B. B. (2012). Brief report: the assessment of anxiety in high-functioning adolescents with autism spectrum disorder. *Journal of Autism and Developmental Disorders,**42*(6), 1138–1145. 21874396 10.1007/s10803-011-1353-3

[CR83] White, S. W., Ollendick, T., Albano, A. M., Oswald, D., Johnson, C., Southam-Gerow, M. A., et al. (2013). Randomized controlled trial: multimodal anxiety and social skill intervention for adolescents with autism spectrum disorder. *Journal of Autism and Developmental Disorders,**43*(2), 382–394. 22735897 10.1007/s10803-012-1577-x PMC3494811

[CR81] Wilkenfeld, D. A., & McCarthy, A. M. (2020). Ethical concerns with applied behavior analysis for autism spectrum “disorder”. *Kennedy Institute of Ethics Journal,**30*(1), 31–69. 10.1353/ken.2020.0000 32336692 10.1353/ken.2020.0000

[CR84] Wood, J. J., Drahota, A., Sze, K., Har, K., Chiu, A., & Langer, D. A. (2009). Cognitive behavioral therapy for anxiety in children with autism spectrum disorders: a randomized, controlled trial. *Journal of Child Psychology and Psychiatry,**50*(3), 224–234. 19309326 10.1111/j.1469-7610.2008.01948.x PMC4231198

[CR85] Wood, J. J., Ehrenreich-May, J., Alessandri, M., Fujii, C., Renno, P., Laugeson, E., et al. (2015). Cognitive behavioral therapy for early adolescents with autism spectrum disorders and clinical anxiety: a randomized, controlled trial. *Behavior Therapy,**46*(1), 7–19. 25526831 10.1016/j.beth.2014.01.002 PMC4272761

